# Do halophytes and glycophytes differ in their interactions with arbuscular mycorrhizal fungi under salt stress? A meta-analysis

**DOI:** 10.1186/s40529-020-00290-6

**Published:** 2020-04-19

**Authors:** Jing Pan, Fei Peng, Anna Tedeschi, Xian Xue, Tao Wang, Jie Liao, Wenjuan Zhang, Cuihua Huang

**Affiliations:** 1 Drylands Salinization Research Station, Key Laboratory of Desert and Desertification, Northwest Institute of Eco-Environment and Resources, Chinese Academy of Sciences, 320 West Donggang Road, Lanzhou, 730000 China; 2 University of Chinese Academy of Sciences, Beijing, 100049 China; 3 International Platform for Dryland Research and Education, Arid Land Research Center, Tottori University, Tottori, 680-0001 Japan; 4 Institute for Agricultural and Forest Mediterranean Systems, National Research Council (CNR) of Italy, Naples, 80056 Italy

**Keywords:** Arbuscular mycorrhizal fungi, Glycophytes, Halophytes, Meta-analysis, Plant growth, Salt stress

## Abstract

**Background:**

Halophytes are better than glycophytes at employing mechanisms to avoid salt injury, but both types of plants can undergo damage due to high soil salinity. Arbuscular mycorrhizal fungi (AMF) can mitigate the damage from salt stress in both halophytes and glycophytes by enhancing salt tolerance and improving energy efficiency. However, variations in mycorrhizal symbiotic efficiency between halophytes and glycophytes were still poorly understood. Therefore, we evaluated the magnitude of AMF effects on plant growth and determined the mechanisms that regulate the growth response of halophytes and glycophytes by performing a meta-analysis of 916 studies (from 182 publications).

**Results:**

Arbuscular mycorrhizal fungi significantly enhance biomass accumulation, osmolytes synthesis (soluble sugar and soluble protein), nutrients acquisition (nitrogen, phosphorus, and potassium ion), antioxidant enzyme activities (superoxide dismutase and catalase), and photosynthetic capacity (chlorophyll and carotenoid contents, photosynthetic rate, stomatal conductance, and transpiration rate). AMF also substantially decreased sodium ion acquisition and malondialdehyde levels in both halophytes and glycophytes under salt stress conditions. Mycorrhizal halophytes deploy inorganic ions (potassium and calcium ions) and limited organic osmolytes (proline and soluble sugar) to achieve energy-efficient osmotic adjustment and further promote biomass accumulation. Mycorrhizal glycophytes depend on the combined actions of soluble sugar accumulation, nutrients acquisition, sodium ion exclusion, superoxide dismutase elevation, and chlorophyll synthesis to achieve biomass accumulation.

**Conclusions:**

Arbuscular mycorrhizal fungi inoculation is complementary to plant function under salt stress conditions, not only facilitating energy acquisition but also redistributing energy from stress defence to growth. Glycophytes are more dependent on AMF symbiosis than halophytes under salt stress conditions.

## Background

Soil salinity is a devastating environmental stress globally that causes severe agronomical and ecological problems, particularly in arid and semiarid regions (Estrada et al. 2013b; Himabindu et al. 2016). From an agronomical standpoint, salinity restricts the area of agricultural land (Sardo and Hamdy 2005), limits the productivity of agricultural crops (Apse 1999), reduces the potential utilization of glycophytes as crops, and impacts livelihood choices and land use strategies (Anik et al. 2018), which further threatens human dietary and food security (Rewald et al. 2013). From an ecological standpoint, salinity decreases plant and microbe abundances (Folli-Pereira et al. 2013), destroys ecosystem diversity (Chaves et al. 2009), accelerates soil and environmental degradation processes (Estrada et al. 2013b), and, consequently, affects the entire food chain. Faster-than-predicted climate change has exacerbated the severity of salt stress (Chaves et al. 2009; Ilangumaran and Smith 2017), and saline soils continue to become more prevalent (Anik et al. 2018). Indeed, the area of land affected by salinity is estimated to be increasing at a rate of 15 to 20 million hectares per year (Sardo and Hamdy 2005), and the economic loss caused by soil salinity is approximately 27.3 billion dollars (Suarez et al. 2015).

Soil salinity initially impairs plants by causing osmotic stress, which induces water deficit and results in physiological drought (Evelin et al. 2009; Munns and Tester 2008). Specific salt ions, such as sodium and chloride ions, cause toxic ionic stress and nutrient deficiency (Munns and Tester 2008; Osman 2018). As a consequence of osmotic and ionic stresses, a series of secondary physiological stresses may occur, such as oxidative damage caused by reactive oxygen species (ROS) (Egamberdieva et al. 2017; Zhu 2001) and photosynthesis limitation (Chen et al. 2017), which synergistically impair plant growth. However, plants respond differently with regard to their tolerance to salinity and can be divided into salt-tolerant halophytes and salt-sensitive glycophytes depending on their different growth adaptations (Kosová et al. 2013; Munns and Tester 2008). Halophytes employ effective salt-tolerance mechanisms to avoid salt damage and stay relatively “calm” (Tester 2003), whereas glycophytes “panic” under salt stress conditions due to limited salt-tolerance mechanisms (Munns and Tester 2008; Zhu 2001). Although the responses of halophytes and glycophytes vary qualitatively and quantitatively under high salt stress, both types of plants will be injured at the early vegetative stage (Himabindu et al. 2016; Munns and Tester 2008).

Arbuscular mycorrhizal fungi (AMF) are beneficial below-ground microbes that form symbiotic relationships with over 80% terrestrial plant species, including halophytes and glycophytes (Avis et al. 2008; Estrada et al. 2013b; Evelin et al. 2009). AMF can improve the growth performance and salinity tolerance of host plants by mediating critical physiological processes, such as facilitating water and nutrient uptake (Alkaraki 2000; Balliu et al. 2015; Chen et al. 2017; Mohammad et al. 2003; Zuccarini 2007), maintaining ion balance (Garg and Bhandari 2016), protecting cells from oxidative damage (He et al. 2007; Yang et al. 2016), and increasing photosynthetic ability (Chen et al. 2017; Estrada et al. 2013b; Sheng et al. 2008). As salt-sensitive crops are closely related to people’s lives and affect food safety, studies about AMF inoculation under salt stress have mainly concentrated on glycophytic crops, such as maize (Estrada et al. 2013a; Liu et al. 2016; Sheng et al. 2008; Wu et al. 2005; Zhang et al. 2018), wheat (Liu 2016; Mardukhi et al. 2011; Talaat and Shawky 2011), tomato (Balliu et al. 2015; He and Huang 2013; Khalloufi et al. 2017; Ouziad et al. 2006), and pepper (Hegazi et al. 2017; Kaya et al. 2009; Turkmen et al. 2008), and so forth.

Recently, a growing number of publications have evaluated interactions between AMF and halophytes under salt stress conditions, including *Asteriscus maritimus* (Estrada et al. 2013b), *Puccinellia tenuiflora* (Liu et al. 2018), *Phragmites australis* (Algarni 2006), for soil phytoremediation and carbon dioxide sequestration in salinized environments (Hasanuzzaman et al. 2015; Sardo and Hamdy 2005). Overall, AMF inoculation efficiency, as determined by different parameters, varies among host plant species with different salt tolerant capabilities (Alkaraki 2001; Ciftci et al. 2010; Fan et al. 2012). However, previous studies have not provided any clues regarding the mechanisms that lead to these differences, which will impede the effective utilization of AMF in agriculture and ecosystem (Folli-Pereira et al. 2013).

Meta-analysis offers a quantitative synthesis method to provide meaningful summaries and uncover new patterns or to reach a consensus among the findings of multiple studies (Hedges et al. 1999; Lehmann and Rillig 2015). Although several scholars have conducted meta-analyses on the effectiveness of AMF on different predictor variables, such as plant types, soil types, AMF inoculums and salinity degrees (Auge et al. 2014; Chandrasekaran et al. 2014, 2016), none have focused explicitly on the effects of AMF inoculation on different salt-tolerant plant species and determined the mechanisms that lead to variation in growth responses. Recently, we conducted a meta-analysis reported that the biomass improvements in salt-sensitive plants were higher than that in salt-tolerant plants after plant growth promoting rhizobacteria (PGPR) inoculation under salt stress conditions, and plant salt tolerance is a determining factor affecting the mechanisms of PGPR promotion (Pan et al. 2019). Do salt-tolerant halophytes and salt-sensitive glycophytes differ in their interaction with AMF under salt stress? Are glycophytes more dependent on mycorrhizal symbiosis than halophytes under salt stress conditions? It is still an enigma. Worthy of mention is that the growing number of Chinese publications gauging interactions between AMF and plants under salt stress conditions, that would often be overlooked in conventional meta-analysis, provides a useful opportunity to apply meta-analysis to resolve this enigma.

Thus, the aim of the present study was to (1) evaluate AMF inoculation efficiency on the biomass accumulation, osmotic adjustment, nutrient acquisition, antioxidative ability, and photosynthetic capacity of both halophytes and glycophytes under salt stress conditions, and (2) uncover the underlying mechanisms of growth promotion in halophytes and glycophytes derived from mutualistic interactions between plants and AMF under salt stress conditions.

## Materials and methods

### Literature search and eligibility criteria

We employed three methods to retrieve relevant publications published before August 2018 for this meta-analysis. We first collected publications from the Web of Science using the keywords as in the methods of previous meta-analyses (Auge et al. 2014; Chandrasekaran et al. 2014, 2016). Meanwhile, we collected publications from the China Knowledge Resource Integrated Database (CNKI) using keyword with Chinese word. We then checked the examined reference list cited in the publications identified from the keyword search. Retrieved publications written in English and Chinese were screened to satisfy the following criteria: (1) The experimental design included parallel control and AMF treatments; (2) Plants were only inoculated with AMF, and there was no interaction with other microbes; (3) Plants were exposed to saline conditions or exposed to salt treatments through irrigation; (4) Plants were grown in pots.

One publication may yield multiple studies, such as different plant species inoculated with different AMF under the same or different salt stresses, which we did not consider to be a violation of independence. Although halophytes are classified in a variety of ways and the plant responses to environmental stresses are very dynamic, plants were only divided into halophytes and glycophytes according to the salt tolerance description in the original publications (see Additional file 1). We hypothesized that salt levels would cause a stress response in the halophytes and glycophytes in the original publication. Finally, 916 studies were extracted from 182 publications from February 1983 to August 2018 describing the effects of AMF inoculation under saline conditions (see Additional files 1, 2).

### Data category and database construction

Salt tolerance has complex traits that involves various mechanisms in plants (Zhang and Shi 2013). Thus, we analyzed the magnitudes and mechanisms of growth promotion in halophytes and glycophytes after AMF inoculation by collecting 21 response parameters, including biomass (total biomass, shoot biomass, and root biomass), nutrient uptake (nitrogen, phosphorus, potassium ion, calcium ion, magnesium ion, and sodium ion), osmolyte accumulation (proline, soluble sugar, soluble protein), antioxidative defense (superoxide dismutase, catalase, and malondialdehyde), and photosynthetic capacity (chlorophyll a, chlorophyll b, carotenoid, net photosynthetic rate, stomatal conductance, and transpiration rate) (Table 1).

**Table 1:** Rank correlation tests and fail-safe numbers for publication bias

Response parameters (Abbreviation)	Study numbers (*S*)	Effect size (95% CI)	Spearman’s rank order correlation		Fail-safe numbers
			R	P	
Total biomass	589	0.29 (0.24 to 0.33)	− 0.041	0.319	2 073 226
Shoot biomass	499	0.39 (0.34 to 0.43)	− 0.133	0.003	2 284 188
Root biomass	423	0.41 (0.35 to 0.47)	− 0.12	0.013	2 380 881
Proline (Pro)	263	− 0.08 (− 0.14 to − 0.02)	− 0.009	0.881	97 751
Soluble sugar (SS)	143	0.18 (0.14 to 0.21)	− 0.195	0.02	143 753
Soluble protein (SP)	104	0.2 (0.17 to 0.24)	0.129	0.192	130 459
Nitrogen (N)	287	0.23 (0.20 to 0.27)	0.006	0.914	483 301
Phosphorus (P)	395	0.38 (0.34 to 0.42)	0.138	0.006	1 637 564
Potassium ion (K^+^)	351	0.24 (0.20 to 0.28)	0.146	0.006	826 281
Calcium ion (Ca^2+^)	166	0.12 (0.06 to 0.18)	− 0.21	0.006	7 106
Magnesium ion (Mg^2+^)	163	0.1 (0.04 to 0.16)	0.191	0.015	20 747
Sodium ion (Na^+^)	307	− 0.17 (− 0.23 to − 0.12)	− 0.098	0.085	363 110
Superoxide dismutase (SOD)	200	0.19 (0.14 to 0.23)	− 0.048	0.502	555 663
Catalase (CAT)	162	0.16 (0.11 to 0.21)	0.099	0.212	56 886
Malondialdehyde (MDA)	170	− 0.24 (− 0.27 to − 0.21)	− 0.028	0.719	708 409
Chlorophyll a (Chla)	102	0.4 (0.32 to 0.49)	− 0.132	0.186	228 892
Chlorophyll b (Chlb)	103	0.33 (0.27 to 0.39)	− 0.033	0.74	152 797
Carotenoid (Car)	59	0.27 (0.18 to 0.37)	0.242	0.064	125 869
Net photosynthetic rate (Pn)	105	0.46 (0.39 to 0.56)	− 0.008	0.938	397 027
Stomatal conductance (Gs)	116	0.47 (0.40 to 0.54)	− 0.02	0.830	486 569
Transpiration rate (Tr)	95	0.48 (0.40 to 0.56)	0.057	0.585	521 965

Means, standard deviations (*SD*), and sample sizes (*n*) in the control and AMF inoculation groups under salt stress or saline conditions were collected from tables in the original publications to build a database. WebPlotDigitizer software was used to estimate values when results were presented graphically (Worchel et al. 2013). Standard errors (*SE*) reported in tables and graphs were all transformed to *SD* according to the equation ($SD = SE \times \sqrt n$) (Chandrasekaran et al. 2016). We also assumed that unidentified error bars represented *SD*.

### Effect size calculations and results analysis

Effect size (*lnR*) is the natural log of response ratio (*R*), which is the ratio of the outcome in the AMF-treated group to that of the control group (Rosenberg et al. 2000). We evaluated the effects of AMF inoculation on halophytes and glycophytes under salt stress by estimating *lnR* and variance (*V*) with a random effect model in the software MetaWin version 2.1.4 (Sinauer Associates, Inc. Sunderland, MA, USA). *lnR* and *V* were calculated using Eqs. 1 and 2 (Hedges et al. 1999; Chandrasekaran et al. 2014), as follows:

1 $$\ln R = \ln \left( {\frac{{\overline{X}_{T} }}{{\overline{X}_{c} }}} \right) = \ln n\left( {\overline{X}_{T} } \right) - \ln \left( {\overline{X}_{C} } \right)$$

2 $$V = \frac{{(S_{T} )^{2} }}{{N_{T} \left( {\overline{X}_{T} } \right)^{2} }} + \frac{{(S_{C} )^{2} }}{{N_{C} \left( {\overline{X}_{C} } \right)^{2} }}$$

where *X*_*T*_, *S*_*T*_, and *N*_*T*_ are the mean, *SD*, and *n* of response parameters with AMF inoculation, respectively. *X*_*C*_, *S*_*C*_, and *N*_*C*_ are the mean, *SD*, and *n* of response parameters without AMF inoculation, respectively. To evaluate AMF inoculation efficiency, back-transformed *lnR* (reported as a percent change under AMF inoculation) was calculated using the equation (exp.(*lnR*) − 1) * 100 (Chandrasekaran et al. 2014). Zero value indicates no difference in the response parameter between the control and AMF inoculation treatments. Positive and negative values indicate an increase and a decrease in the measured parameter, respectively (Auge et al. 2014; Chandrasekaran et al. 2014). To test whether *lnR* was significantly different from zero, a bootstrapping approach with 4999 iterations and implemented bias-correction was used to estimate confidence intervals (95% CI) (Chandrasekaran et al. 2016). The difference between halophytes and glycophytes was compared by P_between_ (*P*_*b*_) associated with Q_between_ statistics (*Q*_*b*_) (Yang et al. 2015).

We performed Spearman correlation analysis and calculated the fail-safe number of Rosenthal methods to examine whether our database was publication-biased using software MetaWin version 2.1 (Chandrasekaran et al. 2016; Pan et al. 2019; Yang et al. 2015). The correlations of *lnR* with different parameters were examined by single regression analysis using SPSS 17.0 software (SPSS for Windows, Version 17.0, Chicago, USA).

## Results

### Overview

The host plants in this meta-analysis belonged to 26 families and included 74 glycophyte species and 14 halophyte species (Additional file 2: Table S1). *Funneliformis mosseae* (35.90%) and *Rhizophagus irregularis* (16.05%) were the major AMF inoculants, accounting for approximately half of all the studies (see Additional file 1 for raw data). The results of Spearman correlation analysis showed significant correlations between *lnR* and *n* for shoot biomass, root biomass, SS, P, K^+^, Ca^2+^, and Mg^2+^ (Table 1), which indicated that slight publication bias for these response parameters. However, the fail-safe numbers of these parameters were much larger than 5*S *+ 10 (*S* indicates studies numbers), which indicated that the publication bias could safely be ignored and would not change the meaning of the results (Table 1).

### Effects of AMF inoculation on biomass in halophytes and glycophytes

**Fig. 1:**
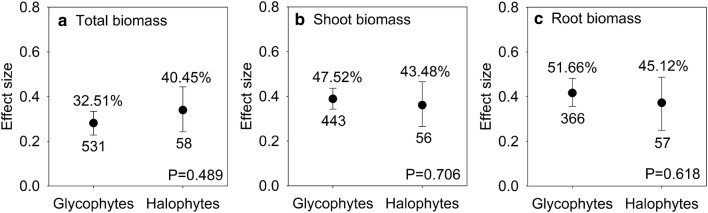
Responses to salt stress of total biomass (**a**), shoot biomass (**b**) and root biomass (**c**) in mycorrhizal halophytes and mycorrhizal glycophytes. Error bars represent 95% confidence intervals (CIs). Back-transformed effect sizes are shown above the bars. The numbers of studies are shown under the bars. Inoculation effects were considered significant when the 95% CIs did not overlap with zero. The P values for each panel reflect the significant differences between halophytes and glycophytes

AMF inoculation significantly increased total biomass, shoot biomass, and root biomass by 33.64% (95% CI, 27.13% to 39.1%), 47.7% (95% CI, 40.5% to 53.73%), and 50.68% (95% CI, 41.91% to 59.9%) compared with levels in control, respectively (Table 1). However, there was no significant difference between mycorrhizal halophytes and mycorrhizal glycophytes in biomass accumulation under salt stress conditions (P > 0.05) (Fig. 1).

### Effects of AMF inoculation on osmolytes in halophytes and glycophytes

**Fig. 2:**
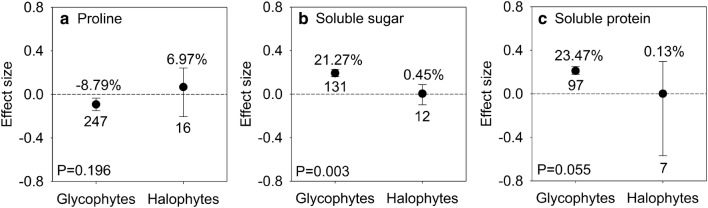
Responses to salt stress of proline (**a**), soluble sugar (**b**) and soluble protein (**c**) in mycorrhizal halophytes and mycorrhizal glycophytes. Error bars represent 95% confidence intervals (CIs). Back-transformed effect sizes are shown above the bars. The numbers of studies are shown under the bars. Inoculation effects were considered significant when the 95% CIs did not overlap with zero. The P values for each panel reflect the significant differences between halophytes and glycophytes

In response to salinity stress, mycorrhizal halophytes and mycorrhizal glycophytes perform differently. Pro (95% CI, − 14.02% to − 3.4%) decreased significantly but SS (95% CI, 17.4% to 25.62%) and SP (95% CI, 19.57% to 28.47%) increased markedly in glycophytes under salt stress. Conversely, no significant *lnR* was observed in Pro (95% CI, − 18.46% to 27.43%), SS (95% CI, − 9.3% to 9.12%), and SP (95% CI, − 43.4% to 34.57%) in halophytes, as the 95% CIs overlapped with zero (Fig. 2).

### Effects of AMF inoculation on nutrient uptake in halophytes and glycophytes

**Fig. 3:**
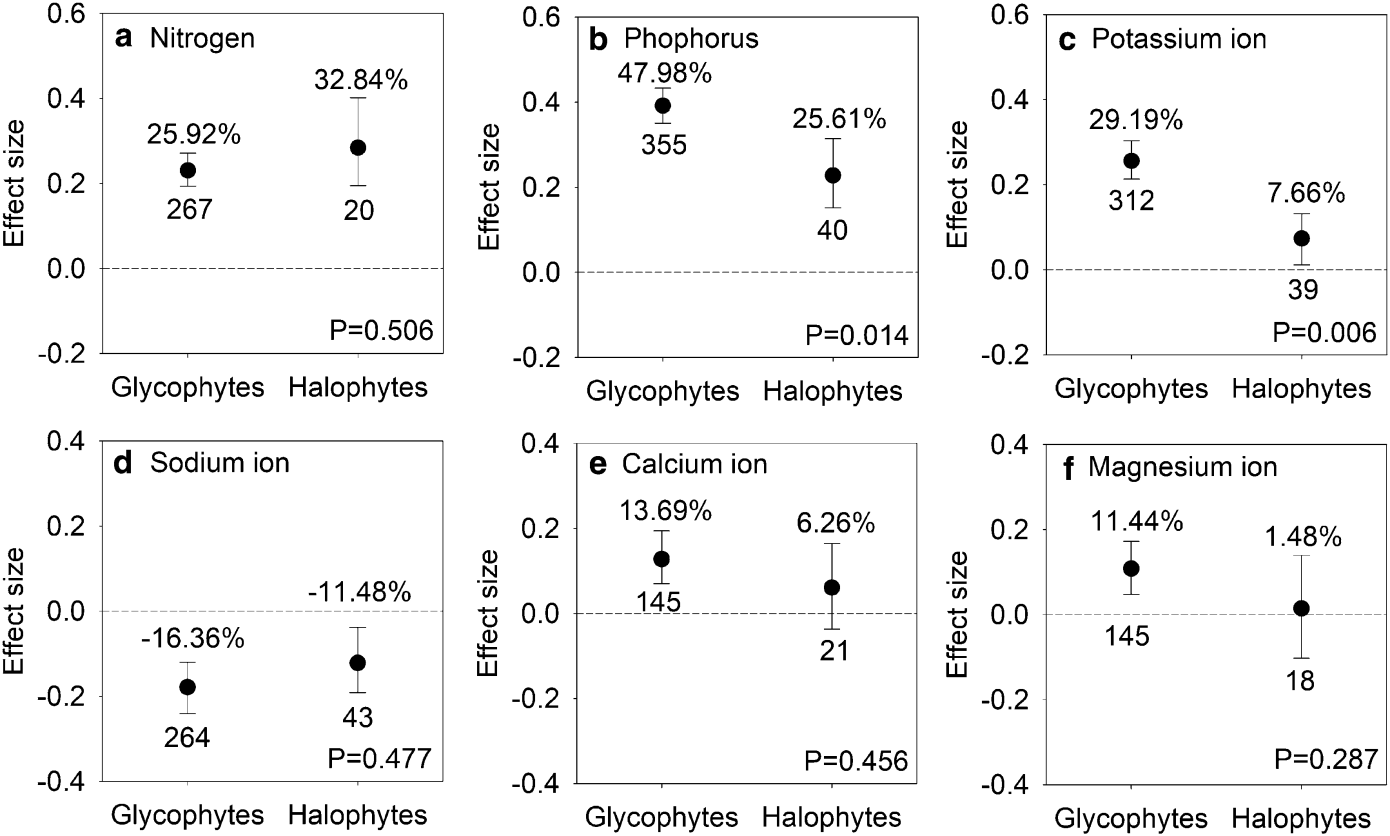
Responses to salt stress of nitrogen (**a**), phosphorus (**b**), potassium ion (**c**), sodium ion (**d**), calcium ion (**e**), and magnesium ion (**f**) in AMF-inoculated halophytes and glycophytes. Error bars represent 95% confidence intervals (CIs). Back-transformed effect sizes are shown above the bars. The numbers of studies are shown under the bars. Inoculation effects were considered significant when the 95% CIs did not overlap with zero. The P values for each panel reflect the significant differences between halophytes and glycophytes

Uptake of N (95% CI, 22.14% to 30.99%), P (95% CI, 40.49% to 52.2%), and K^+^ (95% CI, 22.14% to 32.31%) considerately increased but the uptake of Na^+^ (95% CI, − 20.54% to − 11.31%) remarkedly decreased in plants after AMF inoculation under salt stress conditions (Table 1). There was no significant difference between mycorrhizal halophytes and mycorrhizal glycophytes in N uptake under salt stress conditions (P > 0.05) (Fig. 3a). The *lnR*s of P and K^+^ were higher in glycophytes than in halophytes (P < 0.05) (Figs. 3b, c). Ca^2+^ (95% CI, 7.25% to 21.43%) and Mg^2+^ (95% CI, 4.89% to 18.8%) were only significantly increased in mycorrhizal glycophytes because the 95% CIs did not overlap with zero (Fig. 3e, f).

### Effects of AMF inoculation on antioxidants and MDA in halophytes and glycophytes

**Fig. 4:**
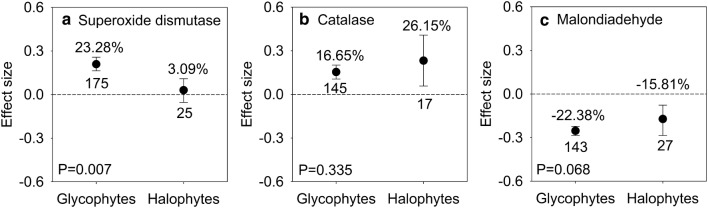
Responses to salt stress of superoxide dismutase (**a**), catalase (**b**) and malondialdehyde (**c**) in mycorrhizal halophytes and mycorrhizal glycophytes. Error bars represent 95% confidence intervals (CIs). Back-transformed effect sizes are shown above the bars. The numbers of studies are shown under the bars. Inoculation effects were considered significant when the 95% CIs did not overlap with zero. The P values for each panel reflect the significant differences between halophytes and glycophytes

Mycorrhizal glycophytes (95% CI, 17.82% to 29.29%) had significantly increased SOD activities, while mycorrhizal halophytes (95% CI, − 5.24% to 11.53%) showed no significant increase (Fig. 4a). Significantly positive impacts on CAT and negative impacts on MDA were observed in both mycorrhizal halophytes and mycorrhizal glycophytes under salt stress, and there was no difference between AMF-inoculated halophytes and AMF-inoculated glycophytes (Fig. 4b, c).

### Effects of AMF inoculation on photosynthesis in halophytes and glycophytes

**Fig. 5:**
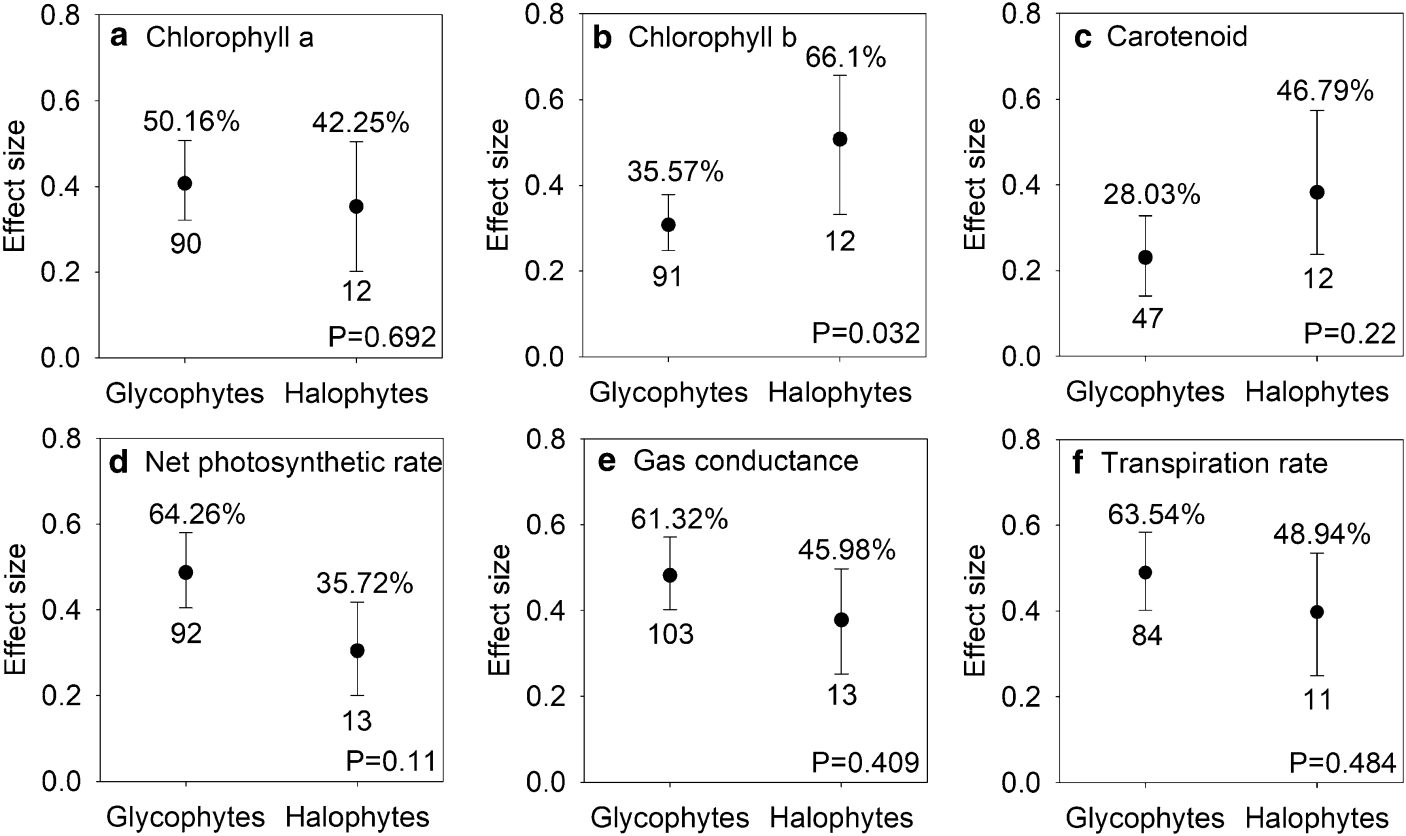
Responses to salt stress of chlorophyll a (**a**), chlorophyll b (**b**), carotenoid (**c**), net photosynthetic rate (d), stomatal conductance (**e**), and transpiration rate (**f**) in mycorrhizal halophytes and mycorrhizal glycophytes. Error bars represent 95% confidence intervals (CIs). Back-transformed effect sizes are shown above the bars. The numbers of studies are shown under the bars. Inoculation effects were considered significant when the 95% CIs did not overlap with zero. The P values for each panel reflect the significant differences between halophytes and glycophytes

AMF inoculation dramatically increased Chla, Chlb, and Car by 49.18% (95% CI, 37.71% to 63.23%), 39.1% (95% CI, 31% to 47.7%), and 30.99% (95% CI, 19.72% to 44.77%), respectively (Table 1). Significant difference was only found in Chlb, mycorrhizal halophytes increased higher than that in mycorrhizal glycophytes under salt stress conditions (Fig. 5b). Pn, Gs, and Tr also significantly increased by 58.41% (95% CI, 47.7% to 75.07%), 59.99% (95% CI, 49.18% to 71.6%), and 61.61% (95% CI, 49.18% to 75.07%) compared with levels in control, respectively (Table 1), but there was no significant difference between mycorrhizal halophytes and mycorrhizal glycophytes under salt stress conditions (Fig. 5d–f).

### Contributions of the different parameters to biomass promotion

**Fig. 6:**
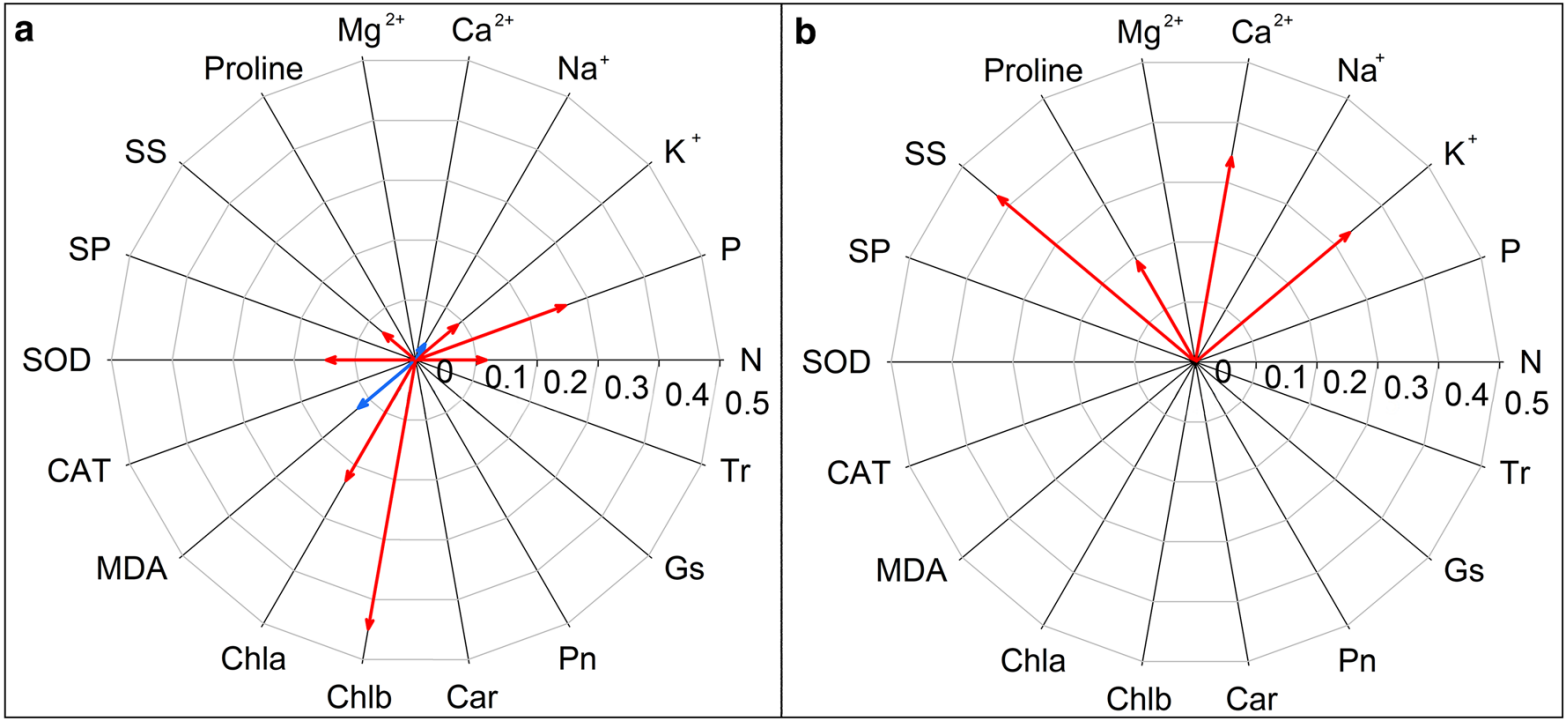
Relationships between the effect size of total biomass and the effect sizes of other physiological indicators for glycophytes (**a**) and halophytes (**b**) under salt stress conditions. Red arrows indicate positive relationships. Blue arrows indicate negative relationships. The arrow length indicates the value of R^2^

The *lnR*s of total biomass correlated positively with the *lnR*s of SS, N, P, K^+^, SOD, Chla, and Chlb whilst negatively correlated with the *lnR*s of Na^+^ and MDA in mycorrhizal glycophytes under salt stress conditions (Fig. 6a). However, positive relationships between *lnR*s of total biomass and *lnR*s of K^+^, Ca^2+^, Pro, and SS were observed in mycorrhizal halophytes under salt stress conditions (Fig. 6b).

## Discussion

Plant salt tolerance is usually quantified as harvestable biomass (Ilangumaran and Smith 2017). The present meta-analysis indicates that AMF significantly elevate biomass accumulation in both halophytes and glycophytes (Fig. 1), which supports the view that AMF can enhance the tolerance of plants to cope with salt stress (Bothe 2012; Evelin et al. 2009; Porcel et al. 2012). AMF confer salt tolerance and fitness in plants by accumulating osmolytes, maintaining ion equilibrium, improving water and nutrient uptake, decreasing oxidative damage, and increasing photosynthetic capacity (Latef and Miransari 2014; Bothe 2012; Evelin et al. 2009). Therefore, we compared the above physiological metabolic processes to uncover the underlying mechanisms of growth promotion in mycorrhizal halophytes and mycorrhizal glycophytes under salt conditions.

### Inoculation effects on osmotic adjustment in halophytes and glycophytes

Soil salinity initially induces osmotic pressure to inhibit water absorption of root and induce water deficit, which immediately further retards the growth of root and shoot (Munns and Tester 2008). Plants cope with osmotic stress mainly due to osmotic adjustment by absorbing soil inorganic ions and accumulating compatible solutes. In general, the utilization of inorganic ions can alleviate physiological drought and maintain osmotic adjustment in plants in an energy-efficient manner (Munns et al. 2016), while compatible solutes are energetically much more expensive (Tester 2003). Halophytes mainly use inorganic ions to efficiently facilitate osmotic adjustment and have a meagre need for organic solutes, while glycophytes primarily use K^+^ and compatible solutes to maintain low osmotic potential (Himabindu et al. 2016; Munns et al. 2016).

The optimization of root morphological structures could help plant to improve ability to absorb and metabolize water (Rewald et al. 2013). Arbuscular mycorrhizal symbiosis could improve the morphological and physiological characteristics of roots, which further helps mycorrhizal plants absorb water and nutrient to prevent the harmful effects of osmotic stress (Gupta and Krishnamurthy 1996; Sheng et al. 2009). Moreover, the hyphae of AMF could extend from the root and bypass the depletion zone of water and nutrient around the root to utilize more soil resources (Tallapragada 2017; Yang et al. 2015). The effects of both root modification and hyphae extension jointly expand the absorbing surface and promote the ability of mycorrhizal plants to take up water and nutrients from soil with low water potential (Elhindi et al. 2017; Gupta and Krishnamurthy 1996; Sheng et al. 2009; Tallapragada 2017; Zou and Wu 2011). In this meta-analysis, AMF inoculation significantly increased root biomass, root length, root surface area, root volume, and root tip numbers (Tables 1, Additional file 2: Table S2). This optimization of morphological structures in the root further bolster both water and K^+^ acquisition in mycorrhizal plants (Fig. 3c). K^+^ is the pivotal inorganic ion that participates in osmotic adjustment, and absorption of K^+^ induces higher root hydraulic conductivity, improves water status, and, consequently, alleviates osmotic stress (Auge et al. 2014; Tallapragada 2017). In the present investigation, the increased K^+^ acquisition is conducive to biomass accumulation (Fig. 6). Therefore, we speculate that utilization of K^+^ in mycorrhizal plants alleviate the physiological drought induced by osmotic stress, efficiently maintain osmotic adjustment, and distribute the energy used for growth to a certain extent.

AMF can also adjust osmotic potential by raising the synthesis of organic osmolytes in host plants, such as Pro, SS, and SP, which consequently facilitate efficient water use in host plants and help to avoid cellular dehydration under salt stress (Latef and Miransari 2014; Auge et al. 2014; Munns and Tester 2008; Ruiz-Lozano et al. 2012; Yang et al. 2014). However, the magnitudes of the AMF effect on osmolytes accumulation vary differently between mycorrhizal halophytes and mycorrhizal glycophytes. Under salt stress conditions, levels of Pro, SS, and SP changed inconspicuously in mycorrhizal halophytes, while accumulation of SS and SP increased notably in mycorrhizal glycophytes (Fig. 2). Inborn salt-tolerant mechanisms might be the cause of the various responses between halophytes and glycophytes. Halophytes mainly utilize inorganic ions to facilitate osmotic adjustment energetically and meanwhile decrease ion toxicity efficiently via adaptative mechanisms. Thus, there is a meagre need for organic solutes in mycorrhizal halophytes. However, compatible solutes are the primarily osmolytes to facilitate osmotic adjustment in glycophytes (Himabindu et al. 2016; Munns et al. 2016). Therefore, mycorrhizal glycophytes accumulate higher levels of SS and SP than do mycorrhizal halophytes to satisfy the demand for osmotic adjustment (Himabindu et al. 2016; Miyama and Tada 2008; Taji et al. 2004).

### Inoculation effects on ions uptake between halophytes and glycophytes

The impacts of ionic stress on the plants occur later than do those of osmotic stress (Munns and Tester 2008). Na^+^ is the most abundant ion released into saline soils (Munns and Tester 2008). High Na^+^ induces K^+^, Ca^2+^, and Mg^2+^ deficiencies and inhibits N and P absorptions (Rewald et al. 2013; Yi̇ldi̇ri̇m et al. 2009). Similar to the previous meta-analyses examining the AMF inoculation under salt stress (Auge et al. 2014; Chandrasekaran et al. 2014), the results of this study that AMF inoculation increased the uptake of N, P, K^+^, Ca^2+^, and Mg^2+^ while decreased that of Na^+^ (Table 1) indicate that mycorrhizal symbiosis might protect mycorrhizal plants from salt toxicity by increasing nutrients absorption and minimizing toxic ion uptake. The increased K^+^/Na^+^ and Ca^2+^/Na^+^ ratios further prove that mycorrhizal symbiosis also compensates for ion disequilibria and facilitates the maintenance of ion homeostasis (Evelin et al. 2009; Yang et al. 2014) (Additional file 2: Table S2). Nonetheless, more positive effects of P and K^+^ were observed in mycorrhizal glycophytes than in mycorrhizal halophytes indicating that the ability of nutrient absorption in the former is stronger than that in the latter under salt stress (Fig. 2b, c). The significant increases in Ca^2+^ and Mg^2+^ only in mycorrhizal glycophytes also demonstrates that glycophytes are more dependent on AMF symbiosis than the halophytes under salt stress conditions (Fig. 2e, f).

In mycorrhizal glycophytes, the significant correlations between total biomass and N, P, K^+^, and Na^+^ showed that AMF promote glycophytes growth by reducing Na^+^ toxicity and enhancing nutrients uptake (Fig. 6a). In mycorrhizal halophytes, although Ca^2+^ is not increased significantly, total biomass correlated with K^+^ and Ca^2+^ (Fig. 6b). The adaptative mechanisms and potential energy costs between halophytes and glycophytes under salt stress might explain this difference. Glycophytes are unable to efficiently exclude Na^+^, and ionic effects dominate over the osmotic effects (Munns and Tester 2008). Thus, energy production is usually redistributed from growth to ionic stress defense (Munns and Tester 2008; Munns and Gilliham 2015). AMF favor biomass accumulation in glycophytes by excluding Na^+^, improving N, P and K^+^ acquisition and reallocating energy from ionic stress defense to growth. Nevertheless, osmotic stress has an immediate effect on the growth of halophytes (Munns and Tester 2008). AMF help halophytes to cope with osmotic stress by deploying the organic solutes and inorganic ions (K^+^ and Ca^2+^), which is useful to maintain efficient ion regulation to satisfy the increased energy demand and metabolism during development (Auge et al. 2014; Himabindu et al. 2016; Munns and Gilliham 2015). Therefore, we suggest that the primary salt tolerance mechanism of mycorrhizal halophytes is the promotion of osmotic adjustment, and the Na^+^ reduction in mycorrhizal halophytes may be the result of a rise in osmotic capacity after AMF inoculation. Regardless, there is limited evidence for this suggestion, and further study is needed.

### Inoculation effects on the antioxidant system between halophytes and glycophytes

Oxidative damage is a secondary effect induced by osmotic and ionic stresses (Zhu 2002). Stress-induced ROS can perturb enzymes, damage cell walls, destabilize membranes and increase MDA contents in plants (Bose et al. 2014; Jithesh et al. 2006). Plants are equipped with enzymatic antioxidants such as SOD and CAT as well as non-enzymatic antioxidants such as Car to scavenge ROS and ultimately minimize salt-induced oxidative damage (Himabindu et al. 2016). Our results showed that AMF significantly upregulated CAT activities and elevated the Car content while reducing the MDA content in both halophytes and glycophytes (Figs. 4b, c, 5c). These results are in accordance with previous studies (Estrada et al. 2013a; He et al. 2007; Yang et al. 2016), and demonstrate the mechanisms by which AMF can induce enzymatic and non-enzymatic antioxidants to alleviate oxidative damage and stabilize membrane (Evelin et al. 2009; Latef and Miransari 2014).

However, mycorrhizal halophytes and glycophytes respond differently to oxidative damage under salt stress conditions. Mycorrhizal glycophytes show higher SOD activity than do mycorrhizal halophytes (Fig. 4a), indicating that antioxidative ability improves more in glycophytes than in halophytes after AMF inoculation. The negative correlations between MDA and SOD as well as CAT in mycorrhizal glycophytes indicate that AMF mitigate oxidative damage mainly by increasing the activities of antioxidative enzymes in glycophytes (Additional file 2: Fig. S1). Furthermore, upregulation of SOD activities and the reductions in MDA contents directly promote the biomass accumulations in mycorrhizal glycophytes (Fig. 6), which indicates that the alleviation of oxidative stress induced by AMF may further promote biomass accumulation in glycophytes under salt stress. In contrast, analogous mechanisms do not appear to function in mycorrhizal halophytes because halophytes have evolved efficient mechanisms to inhibit excessive ROS production in cells at the onset of osmotic and ionic stress (Bose et al. 2014; Himabindu et al. 2016) (Fig. 6). Thus, mycorrhizal halophytes do not need the same level of antioxidants as glycophytes need to mitigate oxidative damage under salt stress conditions.

### Inoculation effects on photosynthetic capacity between halophytes and glycophytes

Photosynthesis is the critical source of energy required for plant growth (Cousins et al. 2014). High salinity induces stomatal closure, reduces photosynthetic capacity, damages photosynthetic pigments synthesis, and ultimately decreases energy acquisition and biomass accumulation (Ashraf and Harris 2013; Chow et al. 1990; Lin et al. 2016; Munns and Gilliham 2015). AMF inoculation significantly increases Chla, Chlb, Car, Pn, Gs, and Tr in both halophytes and glycophytes (Fig. 5), which suggests that AMF might reduce damage to the photosynthetic machinery and enhance photosynthesis capacity in both types of plants under salt stress conditions (Chen et al. 2017). The total chlorophyll content positively correlates with N, P and K^+^ uptake and is negatively associated with MDA (Additional file 2: Fig. S2). This result indicate that the biosynthesis of chlorophyll in mycorrhizal plants is mainly due to both of the increased N, P and K^+^ absorptions (Evelin et al. 2009; Folli-Pereira et al. 2013; Garg and Chandel 2011; Meloni et al. 2001) and the enhanced antioxidant capacities (Hashem et al. 2015; Parida and Das 2005).

Although the magnitudes of AMF on photosynthetic parameters are similar in both halophytes and glycophytes, positive relationships of chlorophyll contents and total biomass occur only in mycorrhizal glycophytes (Fig. 6). The differences of potential energy costs and biomass losses in halophytes and glycophytes might explain this difference. The loss of biomass comprises the losses in both quantity and quality (Rockwood 1984). Biomass quantity can be quantified by dry weight (Meijer and Wijffels 1983), while biomass quality is quantified by chemical components of plants in the ecosystem that can be used as energy and raw materials (Socolow et al. 1996). Glycophytes lack salt-tolerant mechanisms to cope with salt stress, and the limited energy acquired by photosynthesis is mainly used in stress defense under salt stress conditions (Himabindu et al. 2016; Munns and Gilliham 2015). After AMF inoculation, the majority of the increased chlorophyll contents induced by mycorrhizal symbiosis may be redistributed from salt stress defense to the promotion of biomass quantity in glycophytes. Although mycorrhizal halophytes possessed higher photosynthetic capacity and a greater chlorophyll content, there was no correlation between chlorophyll and biomass accumulation (Fig. 6b). It is possible that halophytes have evolved mechanisms to combat salt stress. The energy acquired by photosynthesis is primarily used in general maintenance, and halophytes may invest the majority of the increased chlorophyll contents induced by AMF inoculation to promoting biomass quality (Himabindu et al. 2016; Munns and Gilliham 2015). Therefore, the increased chlorophyll contents are contributed to improve the biomass accumulation in mycorrhizal plants qualitatively and quantitatively, but halophytes and glycophytes lay particular emphasis on each.

## Conclusions

Overall, the results of this meta-analysis suggest that AMF not only enhance plant growth but also alter physiological metabolism processes in plants, such as promoting osmolyte accumulation (SS and SP), nutrient acquisition (N, P, and K^+^), antioxidant enzyme activities (SOD and CAT), and photosynthetic capacity (Chla, Chlb, Car, Pn, Gs and Tr), while decreasing Na^+^-induced damages and MDA contents. Halophytes and glycophytes invoke different growth-promotion mechanisms under salt stress. The growth promotion of mycorrhizal halophytes is mainly due to the energy-efficient improvements in osmoregulation induced by organic osmolytes (Pro and SS) and inorganic ions (K^+^ and Ca^2+^). In contrast, the growth promotion of mycorrhizal glycophytes is achieved via combined actions of accumulating SS, promoting nutrient acquisition, reducing Na^+^ accumulation, enhancing SOD activity and elevating chlorophyll contents. Glycophytes are more dependent on AMF symbiosis than halophytes in nutrient uptake and antioxidant enzymes under salt stress conditions. The inherent salt-tolerance mechanisms in plants are the decisive factors that leads to the different responses in energy acquisition and redistribution between mycorrhizal halophytes and mycorrhizal glycophytes.

## Supplementary information

**Additional file 1.** Raw data and effect sizes in the independent studies in this meta-analysis.

**Additional file 2.** Detailed information of publications, plant salt tolerance classification and plant species in this meta-analysis.

## Data Availability

All data generated or analysed during this study are included in this published article (and its additional information files).

## Abbreviations

AMF: Arbuscular mycorrhizal fungi
Ca^2+^: Calcium ion
Car: Carotenoid
CAT: Catalase
Chla: Chlorophyll a
Chlb: Chlorophyll b
CI: Confidence intervals
Gs: Stomatal conductance
lnR: Effect size
K^+^: Potassium ion
MDA: Malondialdehyde
Mg^2+^: Magnesium ion
N: Nitrogen
Na^+^: Sodium ion
P: Phosphorus
PGPR: Plant growth promoting rhizobacteria
Pn: Net photosynthetic rate
Pro: Proline
ROS: Reactive oxygen species
SOD: Superoxide dismutase
SP: Soluble protein
SS: Soluble sugar
Tr: Transpiration rate
