# Supplementary material for: Do halophytes and glycophytes differ in their interactions with arbuscular mycorrhizal fungi under salt stress? A meta-analysis
Source: Bot Stud. 2020 Apr 19;61:13. doi: 10.1186/s40529-020-00290-6 (PMC7167393; doi:10.1186/s40529-020-00290-6)
Supplement: Supplementary file 2 — Additional file 2. Detailed information of publications, plant salt tolerance classification and plant species in this meta-analysis. [file 40529_2020_290_MOESM2_ESM.docx]

**Title:**

**Do halophytes and glycophytes differ in their interactions with arbuscular mycorrhizal fungi under salt stress? a meta-analysis**

**List of authors**:

Jing Pan^1,2^, Fei Peng^1,3^, Anna Tedeschi^4^, Xian Xue^1^, Tao Wang^1^, Jie Liao^1^, Wenjuan Zhang^1,2^ and Cuihua Huang^1^*

**Affiliations:**

^1^ Drylands Salinization Research Station, Key Laboratory of Desert and Desertification, Northwest Institute of Eco-Environment and Resources, Chinese Academy of Sciences, 320 West Donggang Road, Lanzhou 730000, China

^2^ University of Chinese Academy of Sciences, Beijing 100049, China

^3^ International platform for dryland research and education, Arid Land Research Center, Tottori University, Tottori 680-0001, Japan

^4^ Institute for Agricultural and Forest Mediterranean Systems, National Research Council (CNR) of Italy, Naples 80056, Italy.

* **Corresponding author:** Cuihua Huang (Tel) 86-931- 4967484

(Fax) 86-931-8273894 (E-mail) [hch@lzb.ac.cn](mailto:hch@lzb.ac.cn);

(ORCID): 0000-0003-3413-6531

**Funding:** This research was funded by the National Key Research and Development Program of China (No. 2017YFE0119100), the National Key Research and Development Program of China (No. 2016YFC0500909), and the International Partnership Program of Chinese Academy of Sciences (No. 131B62KYSB20170031).

**References of the studies included in this meta-analysis.**

1. Abdel-Fattah, G. M., & Asrar, A. W. A. (2012). Arbuscular mycorrhizal fungal application to improve growth and tolerance of wheat ( *Triticum aestivum, l.*) plants grown in saline soil. *Acta Physiologiae Plantarum,34* (1):267-277.
2. Abdelfattah G M, Ibrahim A H, Alamri S M, et al. (2013). Synergistic effect of arbuscular mycorrhizal fungi and spermine on amelioration of salinity stress of wheat (*Triticum aestivum L. cv. gimiza 9*). *Australian Journal of Crop Science*, 7(10):1525-1532.
3. Abdel-Rahman SSA, Abdel-Kader AAS, Khalil SE. (2011). Response of Three Sweet Basil Cultivars to Inoculation with Bacillus subtilis and Arbuscular Mycorrhizal Fungi under Salt Stress Conditions. *Nature and Science*, 9: 93-111.
4. Abeer, H., Abd_Allah, E. F., Alqarawi, A. A., Al-Huqail, A. A., Stephan, W., & Dilfuza, E. (2016). The interaction between arbuscular mycorrhizal fungi and endophytic bacteria enhances plant growth ofacacia gerrardiiunder salt stress. *Frontiers in Microbiology*, 7(868).
5. Abeer, H., Abd_Allah, E. F., Alqarawi, A. A., Al-Huqail, A. A., & Shah, M. A. (2016). Induction of osmoregulation and modulation of salt stress inacacia gerrardiibenth. by arbuscular mycorrhizal fungi andbacillus subtilis(bera 71). *Biomed Research International*,(1), 1-11.
6. Algarni, S. M. S. (2006). Increasing NaCl - salt tolerance of a halophytic plant phragmites australis by mycorrhizal symbiosis. *American-Eurasian Journal of Agricultural and Environmental Science*,1(2):119-126.
7. Al-Karaki G N, Hammad R, Rusan M.(2001). Response of two cultivars differing in salt tolerance to inoculation with mycorrhizal fungi under salt stress. *Mycorrhiza*, 11(1):43-48.
8. Al-Karaki G N.(2000). Growth of mycorrhizal tomato and mineral acquisition under salt stress. *Mycorrhiza*, 10(2):51-54.
9. Al-Karaki G N.(2006). Nursery inoculation of tomato with arbuscular mycorrhizal fungi and subsequent performance under irrigation with saline water. *Scientia Horticulturae*, 109(1):1-7.
10. Al-Karaki G N.(2001). Salt stress response of salt-sensitive and tolerant durum wheat cultivars inoculated with mycorrhizal fungi. *Acta Agronomica Hungarica Hungary*, 49(1): 25-36.
11. Al-Khaliel A S.(2001). Effect of salinity stress on mycorrhizal association and growth response of peanut infected by Glomus mosseae. *Plant Soil & Environment*, 56(7): 318-325.
12. Allen, E. B.(1983). Effects of vesicular–arbuscular mycorrhizae on distichlis spicata under three salinity levels. *New Phytologist*, 93(2): 227-236.
13. Alqarawi A A, Allah E F A, Hashem A.(2014). Alleviation of salt-induced adverse impact via mycorrhizal fungi in Ephedra aphylla Forssk. *Journal of Plant Interactions*, 9(1): 802-810.
14. Aroca, R., Porcel, R., & Ruizlozano, J. M. (2010). How does arbuscular mycorrhizal symbiosis regulate root hydraulic properties and plasma membrane aquaporins in phaseolus vulgaris under drought, cold or salinity stresses?. *New Phytologist*, 173(4): 808-816.
15. Aroca, R., Ruizlozano, J. M., Zamarreño, A. M., Paz, J. A., Garcíamina, J. M., & Pozo, M. J., et al. (2013). Arbuscular mycorrhizal symbiosis influences strigolactone production under salinity and alleviates salt stress in lettuce plants. *Journal of Plant Physiology*, 170(1): 47-55.
16. Asghari H R, Marschner P, Smith S E, et al. (2005). Growth response of Atriplex nummularia, to inoculation with arbuscular mycorrhizal fungi at different salinity levels. *Plant & Soil*, 273(1-2): 245-256.
17. Asrar, A. W. A., Abdel-Fattah, G. M., Elhindi, K. M., & Abdel-Salam, E. M. (2014). The impact of arbuscular mycorrhizal fungi in improving growth, flower yield and tolerance of kalanchoe (kalanchoe blossfeldiana poelin) plants grown in nacl-stress conditions. *Journal of Food Agriculture & Environment*, 12(1):105-112.
18. Balliu, A., Sallaku, G., & Rewald, B. (2015). Amf inoculation enhances growth and improves the nutrient uptake rates of transplanted, salt-stressed tomato seedlings.*Sustainability*, 7(12):15967-15981.
19. Barnawal, D., Bharti, N., Maji, D., Chanotiya, C. S., & Kalra, A. (2014). Acc deaminase-containing arthrobacter protophormiae induces nacl stress tolerance through reduced acc oxidase activity and ethylene production resulting in improved nodulation and mycorrhization in pisum sativum. *Journal of Plant Physiology*, 171(11): 884-894.
20. Başak H, Demđr K, Kasim R, et al. (2011). The effect of endo-mycorrhiza (VAM) treatment on growth of tomato seedling grown under saline conditions. *African Journal of Agricultural Research*, 6(11):2532-2538.
21. Bharti N, Barnawal D, Shukla S, et al.(2016). Integrated application of Exiguobacterium oxidotolerans, Glomus fasciculatum, and vermicompost improves growth, yield and quality of Mentha arvensis in salt-stressed soils. *Industrial Crops & Products*, 83:717-728.
22. Bharti N, Barnawal D, Wasnik K, et al.(2016). Co-inoculation of Dietzia natronolimnaea and Glomus intraradices with vermicompost positively influences Ocimum basilicum growth and resident microbial community structure in salt affected low fertility soils. *Applied Soil Ecology*, 100:211-225.
23. Bushra Ghazanfar,et al.(2015).Synergistic and individual effect of glomus etunicatum root colonization and acetyl salicylic acid on root activity and architecture of tomato plants under moderate nacl stress. *Pak. J. Bot*., 47(6):2047-2054.
24. Cantrell I C, Linderman R G. (2001). Preinoculation of lettuce and onion with VA mycorrhizal fungi reduces deleterious effects of soil salinity. *Plant & Soil*, 233(2): 269-290.
25. Chen, J., Zhang, H., Zhang, X., & Tang, M. (2017). Arbuscular mycorrhizal symbiosis alleviates salt stress in black locust through improved photosynthesis, water status, and K^+^/Na^+^ homeostasis. *Frontiers in Plant Science*, 8(8), 1739.
26. Ciftci V., Turkmen, O., Erdi̇Nc, C., & Sensoy, S. (2010). Effects of different arbuscular mycorrhizal fungi (amf) species on some bean (phaseolus vulgaris l.) cultivars grown in salty conditions. *African Journal of Agricultural Research*, 5(24):3408-3416.
27. Ebrahim, M. K. H., & Saleem, A. R. (2017). Alleviating salt stress in tomato inoculated with mycorrhizae: photosynthetic performance and enzymatic antioxidants. *Journal of Taibah University for Science*, 11(6): 850-860.
28. Elhindi K M, Eldin A S, Elgorban A M. (2017). The impact of arbuscular mycorrhizal fungi in mitigating salt-induced adverse effects in sweet basil (Ocimum basilicum L.). *Saudi Journal of Biological Sciences*, 24(1):170.
29. Estrada, B., Aroca, R., Azcón-Aguilar, C., Barea, J. M., & Ruiz-Lozano, J. M. (2013). Importance of native arbuscular mycorrhizal inoculation in the halophyte asteriscus maritimus, for successful establishment and growth under saline conditions. *Plant & Soil*, 370(1-2):175-185.
30. Estrada, Beatriz, Aroca, Ricardo, Barea, M., & Jose, et al. (2013). Native arbuscular mycorrhizal fungi isolated from a saline habitat improved maize antioxidant systems and plant tolerance to salinity. *Plant Science*, 201(1): 42-51.
31. Estrada B, Aroca R, Maathuis F J, et al.(2013). Arbuscular mycorrhizal fungi native from a Mediterranean saline area enhance maize tolerance to salinity through improved ion homeostasis. *Plant Cell & Environment*, 36(10):1771–1783.
32. Evelin, H., Giri, B., & Kapoor, R. (2012). Contribution of glomus intraradices inoculation to nutrient acquisition and mitigation of ionic imbalance in nacl-stressed trigonella foenum-graecum. *Mycorrhiza*,22(3): 203-217.
33. Evelin H, Giri B, Kapoor R.(2013). Ultrastructural evidence for AMF mediated salt stress mitigation in Trigonella foenum-graecum. *Mycorrhiza*, 23(1):71-87.
34. Evelin H, Kapoor R.(2014). Arbuscular mycorrhizal symbiosis modulates antioxidant response in salt-stressed Trigonella foenum-graecum plants. *Mycorrhiza*, 24(3): 197-209.
35. Feng G, Zhang F, Li X, et al.(2002). Improved tolerance of maize plants to salt stress by arbuscular mycorrhiza is related to higher accumulation of soluble sugars in roots. *Mycorrhiza,* 12(4): 185-192.
36. Garg N, Bhandari P. (2016). Silicon nutrition and mycorrhizal inoculations improve growth, nutrient status, K + /Na +, ratio and yield of Cicer arietinum L. genotypes under salinity stress. *Plant Growth Regulation*, 78(3): 371-388.
37. Garg, N., & Manchanda, G. (2009). Role of arbuscular mycorrhizae in the alleviation of ionic, osmotic and oxidative stresses induced by salinity in cajanus cajan (l.) millsp. (pigeonpea). *Journal of Agronomy & Crop Science*, 195(2): 110–123.
38. Garg N, Pandey R. (2016). High effectiveness of exotic arbuscular mycorrhizal fungi is reflected in improved rhizobial symbiosis and trehalose turnover in Cajanus cajan, genotypes grown under salinity stress. *Fungal Ecology*, 21:57-67.
39. Garmendia I, Mangas V J.(2014). Comparative Study of Substrate-Based and Commercial Formulations of Arbuscular Mycorrhizal Fungi in Romaine Lettuce Subjected to Salt Stress. *Journal of Plant Nutrition*, 37(11):1717-1731.
40. Ghazanfar, B., Cheng, Z., Cuinan, W. U., Liu, H., Hezi, L. I., & Rehman, R. N. U., et al. (2016). Glomus etunicatum root inoculation and foliar application of acetyl salicylic acid induced nacl tolerance by regulation of nac1 & lenhxl gene expression and improved photosynthetic performance in tomato seedlings. *Pak. J. Bot*., 48(3): 1209-1217.
41. Ghazi N. Al-Karaki, & R. Hammad. (2001). Mycorrhizal influence on fruit yield and mineral content of tomato grown under salt stress. *Journal of Plant Nutrition*, 24(8):1311-1323.
42. Giri B, Kapoor R, Mukerji K G.(2007). Improved tolerance of Acacia nilotica to salt stress by Arbuscular mycorrhiza, Glomus fasciculatum may be partly related to elevated K/Na ratios in root and shoot tissues. *Microbial Ecology*, 54(4): 753-760.
43. Giri B, Kapoor R, Mukerji K G.(2003). Influence of arbuscular mycorrhizal fungi and salinity on growth, biomass, and mineral nutrition of Acacia auriculiformis. *Biology and Fertility of Soils*, 38(3): 170-175.
44. Giri B, Mukerji K.(2004). Mycorrhizal inoculant alleviates salt stress in Sesbania aegyptiaca and Sesbania grandiflora under field conditions evidence for reduced sodium and improved magnesium uptake. *Mycorrhiza*,14(5): 307-312.
45. Hartmond U, Schaesberg N V, Graham J H, et al. (1987). Salinity and flooding stress effects on mycorrhizal and non-mycorrhizal citrus rootstock seedlings. *Plant & Soil*, 104(1): 37-43.
46. Hashem A, Abd_Allah E F, Alqarawi A A, et al.(2016). Comparing symbiotic performance and physiological responses of two soybean cultivars to arbuscular mycorrhizal fungi under salt stress[J]. *Saudi Journal of Biological Sciences*.
47. Hashem A, Abdallah E F, Alqarawi A A, et al. Arbuscular mycorrhizal fungi enhances salinity tolerance of Panicum turgidum Forssk by altering photosynthetic and antioxidant pathways[J]. Journal of Plant Interactions, 2015
48. Hashem A, Elsayed Fathi Abd Allah, Alqarawi A A, et al.(2016). Arbuscular mycorrhizal fungi alleviate salt stress in lupine (Lupinus termis Forsik) through modulation of antioxidant defense systems and physiological traits. Legume Research, 39(2):198-207.
49. Hashem, A., Alqarawi, A. A., Radhakrishnan, R., Al-Arjani, A. B. F., Aldehaish, H. A., & Egamberdieva, D., et al. (2018). Arbuscular mycorrhizal fungi regulate the oxidative system, hormones and ionic equilibrium to trigger salt stress tolerance in cucumis sativus, l. *Saudi Journal of Biological Sciences,*25:1102–1114.
50. He, Z., He, C., Zhang, Z., Zou, Z., & Wang, H. (2007). Changes of antioxidative enzymes and cell membrane osmosis in tomato colonized by arbuscular mycorrhizae under nacl stress. *Colloids & Surfaces B Biointerfaces*, 59(2): 128-133.
51. He Z, Huang Z. (2013). Expression analysis of LeNHX1 gene in mycorrhizal tomato under salt stress[J]. *Journal of Microbiology,* 51(1):100-104.
52. Hegazi, A. M., El-Shraiy, A. M., & Ghoname, A. A. (2017). Erratum to: mitigation of salt stress negative effects on sweet pepper using arbuscular mycorrhizal fungi (amf), bacillus megaterium, and brassinosteroids (brs). *Gesunde Pflan*zen, 69(2), 111-111.
53. Huang Zhi, He Chao-xing, He Zhong-qun, et al. (2010). The effects of arbuscular mycorrhizal fungi on reactive oxyradical scavenging system of tomato under salt tolerance. *Journal of Integrative Agriculture*, 09(8):1150-1159.
54. Jahromi F, Aroca R, Porcel R, et al.(2008). Influence of Salinity on the In Vitro Development of Glomus intraradices and on the In Vivo Physiological and Molecular Responses of Mycorrhizal Lettuce Plants. *Microbial Ecology*, 55(1):45-54.
55. Kadian N, Yadav K, Badda N, et al.(2013). AM fungi ameliorates growth, yield and nutrient uptake in Cicer arietinum, L. Under salt stress. *Russian Agricultural Sciences*, 39(4):321-329.
56. Kaya C, Ashraf M, Sonmez O, et al.(2009). The influence of arbuscular mycorrhizal colonisation on key growth parameters and fruit yield of pepper plants grown at high salinity. *Scientia Horticulturae*, 121(1):1-6.
57. Khaliel A S, Shine K, Vijayakumar K.(2011). Salt tolerance and mycorrhization of Bacopa monneiri grown under sodium chloride saline conditions. *African Journal of Microbiology Research*, 5(15): 2034-2040
58. Khalloufi M, Martínezandújar C, Lachaâl M, et al.(2017). The interaction between foliar GA3 application and arbuscular mycorrhizal fungi inoculation improves growth in salinized tomato (Solanum lycopersicum L.) plants by modifying the hormonal balance. *Journal of Plant Physiology*, 214: 134-144.
59. Krishnamoorthy R, Kim K, Subramanian P, et al.(2016). Arbuscular mycorrhizal fungi and associated bacteria isolated from salt-affected soil enhances the tolerance of maize to salinity in coastal reclamation soil. *Agriculture Ecosystems & Environment*, 231:233-239.
60. Kumar A, Sharma S, Mishra S.(2009). Effect of alkalinity on growth performance of Jatropha curcas inoculated with PGPR and AM fungi. *Journal of Phytology*, (3):177-184.
61. Kumar, A., Sharma, S., & Mishra, S. (2010). Influence of arbuscular mycorrhizal (am) fungi and salinity on seedling growth, solute accumulation, and mycorrhizal dependency of jatropha curcas l. *Journal of Plant Growth Regulation,* *29*(3), 297-306.
62. Latef A A H A, He C.(2011). Effect of arbuscular mycorrhizal fungi on growth, mineral nutrition, antioxidant enzymes activity and fruit yield of tomato grown under salinity stress. *Scientia Horticulturae*, 127(3): 228-234.
63. Lin J, Wang Y, Sun S, et al. (2017). Effects of arbuscular mycorrhizal fungi on the growth, photosynthesis and photosynthetic pigments of Leymus chinensis seedlings under salt-alkali stress and nitrogen deposition. *Science of the Total Environment*, 576:234-241.
64. Lin, J., Peng, X., Hua, X., Sun, S., Wang, Y., & Yan, X. (2018). Effects of arbuscular mycorrhizal fungi on leymus chinensis seedlings under salt–alkali stress and nitrogen deposition conditions: from osmotic adjustment and ion balance. *Rsc Advances*, 8(26), 14500-14509.
65. Liu C, Dai Z, Cui M, et al. (2018). Arbuscular mycorrhizal fungi alleviate boron toxicity in Puccinellia tenuiflora, under the combined stresses of salt and drought. *Environmental Pollution*, 240:557–565.
66. Liu, S., Guo, X., Feng, G., Maimaitiaili, B., Fan, J., & He, X. (2016). Indigenous arbuscular mycorrhizal fungi can alleviate salt stress and promote growth of cotton and maize in saline fields. *Plant & Soil*, 398(1-2):195-206.
67. Mentis, M., & Lundgren, K. (2011). Growth photosynthetic activity and antioxidant responses of mycorrhizal and non-mycorrhizal bajra (pennisetum glaucum) crop under salinity stress condition. *Crop Protection*, 30(3): 265-271.
68. Mardukhi B, Rejali F, Daei G, et al.(2011). Arbuscular mycorrhizas enhance nutrient uptake in different wheat genotypes at high salinity levels under field and greenhouse conditions. *Comptes Rendus Biologies*, 334(7):564-571.
69. Meca E, Sallaku G, Balliu A.(2016). Could the artificial inoculation of AM fungi improve the benefits of using pea (Pissum sativum L) plants for soil amendment purposes in greenhouses?[C]// III International Symposium on Organic Greenhouse Horticulture, Izmir, Turkey:11-14.
70. Mergulhão A C, Burity H A, Tabosa J N, et al.(2002). Salt stress response and proline accumulation in Brachiaria humidicola plants with and without mycorrhizal inoculation. *Revista Argentina De Microbiologia*, 34(2):77-82.
71. Mirzaei1.J and Moradi.M.(2016). Single and Dual Arbuscular Mycorrhiza Fungi Inoculumn Effects on Growth, Nutrient Absorption and Antioxidant Enzyme Activity in Ziziphus spina-christi Seedlings under Salinity Stress. *J. Agr. Sci. Tech*.,18: 1845-1857.
72. Munir J. Mohammad, Hanan I. Malkawi, Rida Shibli. (2003). Effects of Arbuscular Mycorrhizal Fungi and Phosphorus Fertilization on Growth and Nutrient Uptake of Barley Grown on Soils with Different Levels of Salts. *Journal of Plant Nutrition*, 26(1):125-137.
73. Muok B O, Ishii T. (2006). Effect of Arbuscular Mycorrhizal Fungi on Tree Growth and Nutrient Uptake of Sclerocarya birrea under Water Stress, Salt Stress and Flooding. *Engei Gakkai Zasshi*, 75(75):26-31
74. Murkute A A, Sharma S, Singh S K.(2006). Studies on salt stress tolerance of citrus rootstock genotypes with arbuscular mycorrhizal fungi. *Horticultural Science - UZPI (Czech Republic),* 33(2):70-79.
75. Na W, Zhen L, Liu H, et al. (2015). Influence of arbuscular mycorrhiza on photosynthesis and water status of Populus cathayana, Rehder males and females under salt stress. *Acta Physiologiae Plantarum*, 37(9):183.
76. Navarro J M, Pérez-Tornero O, Morte A. (2014). Alleviation of salt stress in citrus seedlings inoculated with arbuscular mycorrhizal fungi depends on the rootstock salt tolerance. *Journal of Plant Physiology*,171(1):76-85.
77. Oliveira D F B D, Endres L, Silva J V, et al.(2017). Pre-colonized seedlings with arbuscular mycorrhizal fungi: an alternative for the cultivation of Jatropha curcas, L. in salinized soils. *Theoretical & Experimental Plant Physiology*, 29(3):129-142.
78. Önder Türkmen, Çiftçi, V., Çeknas Erdinç, & Şensoy, S. (2010). Effects of Different AMF Species on Some Bean Cultivars Grown in Salty Conditions. *International Symposium on Sustainable Development*, 6: 8-9.
79. Ouziad, F., Wilde, P., Schmelzer, E., Hildebrandt, U., & Bothe, H. (2006). Analysis of expression of aquaporins and Na + /H +, transporters in tomato colonized by arbuscular mycorrhizal fungi and affected by salt stress. *Environmental & Experimental Botany*, 57(1–2):177-186.
80. Paolo Zuccarini, Paulina Okurowska.(2008). Effects of Mycorrhizal Colonization and Fertilization on Growth and Photosynthesis of Sweet Basil Under Salt Stress. *Journal of Plant Nutrition*, 31(3):497-514.
81. Peng, J., Li, Y., Shi, P., Chen, X., Lin, H., & Zhao, B. (2011). The differential behavior of arbuscular mycrorrhizal fungi in interaction with astragalus sinicus l. under salt stress. *Mycorrhiza*, 21(1), 27-33.
82. Rabie G H, Aboul-Nasr M B, Al-Humiany A.(2005). Increased Salinity Tolerance of Cowpea Plants by Dual Inoculation of an Arbuscular Mycorrhizal FungusGlomus clarumand a Nitrogen-fixerAzospirillum brasilense. *Mycobiology*, 33(1): 51-61.
83. Ruedapuente, E. O., Murilloamador, B., Castellanoscervantes, T., Garcíahernández, J. L., Tarazònherrera, M. A., & Moreno Medina, S., et al. (2010). Effects of plant growth promoting bacteria and mycorrhizal on capsicum annuum l. var. aviculare ([dierbach] d'arcy and eshbaugh) germination under stressing abiotic conditions. *Plant Physiology & Biochemistry*, 48(8), 724-736.
84. Ruiz-Lozano J M, Azcón R, Gómez M.(1996). Alleviation of salt stress by arbuscular-mycorrhizal Glomus, species in Lactuca sativa plants. *Physiologia Plantarum*, 98(4): 767-772.
85. Ruiz-Lozano J M, Azcón R.(2000). Symbiotic efficiency and infectivity of an autochthonous arbuscular mycorrhizal Glomus sp. from saline soils and G. deserticola under salinity. *Mycorrhiza*, 10(3):137-143.
86. Sarwat M, Hashem A, Ahanger M A, et al.(2016). Mitigation of NaCl Stress by Arbuscular Mycorrhizal Fungi through the Modulation of Osmolytes, Antioxidants and Secondary Metabolites in Mustard (Brassica juncea L.) Plants. *Frontiers in Plant Science*, 7(868).
87. Scagel, C. F., Bryla, D. R., & Lee, J. (2017). Salt exclusion and mycorrhizal symbiosis increase tolerance to nacl and cacl2 salinity in 'siam queen' basil. *Hortscience A Publication of the American Society for Horticultural Science*, 52(2), 278-287.
88. Selvakumar G, Thamizhiniyan P. (2011).The Effect of the Arbuscular Mycorrhizal (AM) Fungus Glomus intraradices on the Growth and Yield of Chilli (Capsicum annuum L.) Under Salinity Stress[J]. *World Appl Sci J*, 14 (8): 1209-1214.
89. Shamshiri M H, Fattahi M.(2016). Effects of arbuscular mycorrhizal fungi on photosystem II activity of three pistachio rootstocks under salt stress as probed by the OJIP-test. *Russian Journal of Plant Physiology*, 63(1):101-113.
90. Shamshiri, M. H., & Fattahi, M. (2014). Evaluation of two biochemical markers for salt stress in three pistachio rootstocks inoculated with arbuscular mycorrhiza (glomus mosseae). *Journal of Stress Physiology & Biochemistry*, 10(1), 335-346.
91. Sharifi, M., Ghorbanli, M., & Ebrahimzadeh, H. (2007). Improved growth of salinity-stressed soybean after inoculation with salt pre-treated mycorrhizal fungi. *Journal of Plant Physiology*, 164(9), 1144-1151.
92. Sheng, M., Tang, M., Chen, H., Yang, B., Zhang, F., & Huang, Y. (2008). Influence of arbuscular mycorrhizae on photosynthesis and water status of maize plants under salt stress. *Mycorrhiza*, 18(6-7), 287-296.
93. Sheng M, Tang M, Zhang F, et al.(2012). Influence of arbuscular mycorrhiza on organic solutes in maize leaves under salt stress. *Mycorrhiza,* 21(5): 423-430.
94. Sinclair, G. (2013). Influence of colonization by arbuscular mycorrhizal fungi and a root endophyte on selected strawberry cultivars under salt conditions, University of Ottawa.
95. Talaat N B, Shawky B T.(2011). Influence of arbuscular mycorrhizae on yield, nutrients, organic solutes, and antioxidant enzymes of two wheat cultivars under salt stress. *Journal of Plant Nutrition and Soil Science*, 174(2): 283-292.
96. Taliha Uysal Boyacıoglu, & Refik Uyanöz. (2014). Effects of mycorrhizal fungi on tolerance capability of corn grown under salt stress condition. *Journal of Plant Nutrition*, 37(1), 107-122.
97. Tian C Y, Feng G, Li X L, et al.(2004). Different effects of arbuscular mycorrhizal fungal isolates from saline or non-saline soil on salinity tolerance of plants. *Applied Soil Ecology*, 26(2):143-150.
98. Turkmen O, Sensoy S, Demir S, et al. Turkmen O, Sensoy S, Demir S, et al.(2008). Effects of two different AMF species on growth and nutrient content of pepper seedlings grown under moderate salt stress. *African Journal of Biotechnology*,7(4):392-396.
99. Upreti K K, Bhatt R M, Panneerselvam P, et al.(2015). Morpho-Physiological Responses of Grape Rootstock â€˜Dogridgeâ€™ to Arbuscular Mycorrhizal Fungi Inoculation Under Salinity Stress[J]. *International Journal of Fruit Science*, 16(2):191-209.
100. Wang Y, Wang M, Li Y, et al.(2018). Effects of arbuscular mycorrhizal fungi on growth and nitrogen uptake of Chrysanthemum morifolium under salt stress. *Scientific Reports*, 7(1):12181.
101. Wu, Q. S., Zou, Y. N., & He, X. H. (2010). Contributions of arbuscular mycorrhizal fungi to growth, photosynthesis, root morphology and ionic balance of citrus seedlings under salt stress. *Acta Physiologiae Plantarum*, 32(2), 297-304.
102. Wu Q S, Zou Y N, He X H.(2013). Mycorrhizal symbiosis enhances tolerance to NaCl stress through selective absorption but not selective transport of K+ over Na+ in trifoliate orange. *Scientia Horticulturae*, 160: 366-374.
103. Wu Q S, Zou Y N, Liu W, et al.(2010). Alleviation of salt stress in citrus seedlings inoculated with mycorrhiza changes in leaf antioxidant defense systems.*Plant Soil & Environment*, 56(10): 470-475.
104. Wu, Q. S., & Zou, Y. N. (2013). Mycorrhizal symbiosis alters root h+ effluxes and root system architecture of trifoliate orange seedlings under salt stress. *Journal of Animal & Plant Sciences*, 23(1):143-148.
105. Xu HW,Lu Y, Tong S. (2018). Effects of arbuscular mycorrhizal fungi on photosynthesis and chlorophyll fluorescence of maize seedlings under salt stress. *Emirates Journal of Food and Agriculture*, 30(3):199-204.
106. Xueming Zai, Yu Zai, Huanshi Zhang, & Zhenping Hao. (2015). Characterising the rhizospheric soil niches of beach plum (prunus maritima) colonised by arbuscularmycorrhizal fungi and/or phosphate-solubilising fungi when grown under nacl stress. *Journal of Pomology and Horticultural Science*, 90(4):469-475.
107. Xueming, Z., Hao, Z. P., Yu, Z., Zhang, H. S., & Pei, Q. (2014). Arbuscular mycorrhizal fungi (amf) and phosphate-solubilizing fungus (psf) on tolerance of beach plum (prunus maritima) under salt stress. *Australian Journal of Crop Science*, 8(6):945-950.
108. Yamato M, Ikeda S, Iwase K.(2018). Community of arbuscular mycorrhizal fungi in a coastal vegetation on Okinawa island and effect of the isolated fungi on growth of sorghum under salt-treated conditions. *Mycorrhiza*, 18(5):241-252.
109. Yang S J, Zhang Z L, Xue Y X, et al.(2014). Arbuscular mycorrhizal fungi increase salt tolerance of apple seedlings. *Botanical Studies*, 55(1):1-7.
110. Yang, X., H. YU, Y. U., Zhang, T., Guo, J., & Zhang, X. (2016). Arbuscular mycorrhizal fungi improve the antioxidative response and the seed production of suaedoideae species suaeda physophora pall under salt stress. *Notulae Botanicae Horti Agrobotanici Cluj-Napoca*, 44(2), 533-540.
111. Yano-Melo A M, Jr O J S, Maia L C.(2003). Tolerance of mycorrhized banana (Musa sp. cv. Pacovan) plantlets to saline stress. *Agriculture Ecosystems & Environment*, 95(1):343-349.
112. Zhang H, Wu X, Li G, et al.(2011). Interactions between arbuscular mycorrhizal fungi and phosphate-solubilizing fungus (Mortierella sp.) and their effects on Kostelelzkya virginica growth and enzyme activities of rhizosphere and bulk soils at different salinities. *Biology & Fertility of Soils*, 47(5):543-554.
113. Zhang Y C, Wang P, Wu Q H, et al.(2016). Arbuscular mycorrhizas improve plant growth and soil structure in trifoliate orange under salt stress. *Archives of Agronomy & Soil Science*, 63(4):491-500.
114. Zhang, Y. F., Wang, P., Yang, Y. F., Bi, Q., Tian, S. Y., & Shi, X. W. (2011). Arbuscular mycorrhizal fungi improve reestablishment of leymus chinensis in bare saline-alkaline soil: implication on vegetation restoration of extremely degraded land. *Journal of Arid Environments*, 75(9), 773-778.
115. Zhang, W., Wang, C., Lu, T., & Zheng, Y. (2018). Cooperation between arbuscular mycorrhizal fungi and earthworms promotes the physiological adaptation of maize under a high salt stress. *Plant & Soil*, 423(1-2), 125-140.
116. Zhu RF,Tanf FL,Lin JL,et al.(2016).Co-inoculation of arbusculr mycorrhizae and nitrogen fixing bacteria enhance alfalfa yield under saline conditions. *Pak. J. Bot.*, 48(2): 763-770.
117. Zhu, X. Q., Tang, M., & Zhang, H. Q. (2016). Arbuscular mycorrhizal fungi enhanced the growth, photosynthesis, and calorific value of black locust under salt stress. *Photosynthetica*, 1-8.
118. Zhu X, Song F, Liu S, et al. (2016). Role of Arbuscular Mycorrhiza in Alleviating Salinity Stress in Wheat (Triticum aestivum L.) Grown Under Ambient and Elevated CO^2^. *Journal of Agronomy & Crop Science*, 202(6):486-496.
119. Zou Y N, Wu Q S.(2011). Efficiencies of five arbuscular mycorrhizal fungi in alleviating salt stress of trifoliate orange. *International Journal of Agriculture & Biology*, 13(6):991-995.
120. Zuccarini P.(2007). Mycorrhizal infection ameliorates chlorophyll content and nutrient uptake of lettuce exposed to saline irrigation. *Plant Soil & Environment*, 53(53): 281-287.
121. Biqi. (2006).Analysis on Arbuscular Mycorrhizal Fungi to salt-tolerance and growth effects of Leymus chinensis.*Northeast Normal University*.Master dissertation.(With English abstract. 毕琪. 丛枝菌根真菌对羊草耐盐性及生长效应分析[D]. 东北师范大学, 2006.)
122. Cao Yanpo, Dai Peng, Dai Suying, etal.(2015). Effects of arbuscular mycorrhizal fungi(AMF) on seedling grwoth and Na^+^,K^+^, Ca^2+^,Mg^2+^ contents and distribution in asparagus under salt stresss. *Chinese journal of ecology*.34(6)1699-1704. (With English abstract. 曹岩坡, 代鹏, 戴素英,等. 丛枝菌根真菌(AMF)对盐胁迫下芦笋幼苗生长及体内Na^+^、K^+^、Ca^2+^、Mg^2+^含量和分布的影响[J]. 生态学杂志, 2015, 34(6)1699-1704.)
123. Cao Yanpo, Dai Peng, Dai Suying, etal.(2017).Effects of Arbuscular Mycorrhiza Fungi (AMF) on Osmoregulation Substances and Antioxidant Enzyme Activities of Asparagus Plant Under Salt Stress. *Journal of Southwest University (Natural Science Edition)*, 39(5):43-48.(With English abstract. 曹岩坡, 代鹏, 戴素英. 丛枝菌根真菌(AMF)对盐胁迫下芦笋植株渗透调节物质及抗氧化酶活性的影响[J]. 西南大学学报(自然科学版), 2017, 39(5):43-48.)
124. Zeng Guangping, Zhang Xia, Liu Hongling, et al.(2011). Effect of AM Fungi on Salt Tolerance of Carthamus tinctorius L. under Salt stress. *Plant physiology Journal*. 47(11):1069-1074.(With English abstract. 曾广萍, 张霞, 刘红玲,等. 盐胁迫下AM真菌对红花耐盐性的影响[J]. 植物生理学报, 2011, 47(11)1069-1074.)
125. Chen Danming.2010. Effects of Arbuscular Mycorrhizal Fungi on Salt Tolerance of Paeonia suffruticosa. *Qingdao Agricultural University*. Doctoral dissertation (With English abstract. 陈丹明. (2010). 丛枝菌根真菌对牡丹耐盐性的影响. Doctoral dissertation, 青岛农业大学).
126. Chen Xin.2009. Study on Effects of Arbuscular Mycorrhizal Fungi on The Salt and Drought Resistance of Arctium lappa L. *Heilongjiang Bayi Agricultural University*, Master dissertation.(With English abstract. 陈鑫. 丛枝菌根真菌对牛蒡幼苗耐盐性和抗旱性的影响[D]. 黑龙江八一农垦大学, 2009.)
127. Fan Li.2011. Studies on improvement of salt tolerance and fruit quality by arbuscular mycorrhizal fungi inoculation on *strawberry*. *Chinese Academy of Agricultural Sciences Dissertation*.(With English abstract.樊丽. 丛枝菌根真菌对草莓耐盐性及果实品质的影响[D]. 中国农业科学院, 2011.)
128. Feng Gu, Bai Dengsha, Yang Maoqiu,et al. 2000.Influence of inoculating arbuscular mycorrhizal fungi on growh and salinity tolerance parameters of maize plants. *Acta agronomica sinica*, 26(6): 743-750. (With English abstract.冯固, 白灯莎, 杨茂秋,等. 盐胁迫下AM真菌对玉米生长及耐盐生理指标的影响[J]. 作物学报, 2000, 26(6)743-750.)
129. Feng Gu, Li Xiaolin, Zhang Fusuo, et al. (2000).Effect of AM fungi on water and nutrition status of corn plants under salt stress.*Chinese journal of applied ecolocy*,11(4):595-598.(With English abstract.冯固, 李晓林, 张福锁,等. 盐胁迫下丛枝菌根真菌对玉米水分和养分状况的影响[J]. 应用生态学报, 2000, 11(4):595-598.)
130. Feng Gu, Yang Maoqiu.1998. Influence of VAM Fungi onMineral Elements Concentration and Composition in Bromus inermis under Salinity Stress. *Acta prataculturae sinica*, 1998 (3), 21-29. (With English abstract.冯固, 杨茂秋. 盐胁迫下VA菌根真菌对无芒雀麦体内矿质元素含量及组成的影响[J]. 草业学报, 1998(3), 21-29.)
131. Feng Gu, Zhang Suofu. 2003.Effect of arbuscular mycorrhizal fungi on salinity tolerance of cotton. *Chinese journal of eco-agriculture*, 11(2): 21-24.(With English abstract.冯固, 张福锁. 丛枝菌根真菌对棉花耐盐性的影响研究[J]. 中国生态农业学报, 2003, 11(2)21-24.)
132. Feng Xihuan, Liu Weixin, Li Min. 2016. Effects of arbuscular mycorrhizal fungi on growth and physiological indices of Lettuce under salt stress. *Journal of Qiangdao agricultural university (Nature Science)*, 33(4): 242-246. (With English abstract. 冯希环, 刘维信, 李敏. 盐胁迫下丛枝菌根真菌对生菜生长和生理特性的影响[J]. 青岛农业大学学报(自然科学版), 2016, 33(4):242-246.)
133. Gao Chong. 2013.Effects of arbuscular mycorrhizal fungi on the growth and salt tolerance of Pyrus betulaefolia seedling.*Northwest university*. Master dissertation.(With English abstract. 高崇. 接种AM真菌对盐胁迫下杜梨实生苗生长及耐盐性的影响研究[D]. 西南大学, 2013)
134. Guo Jiangyuan, Guo Wei, Bi Na, et al. 2015. Effects of arbuscular mycorrhizal fungi on the growth of Reeds in wetland soils with different salt content. *Envieronmental Science*, 36(4): 1481-1488. (With English abstract. 郭江源, 郭伟, 毕娜,等. 丛枝菌根真菌对不同含盐量湿地土壤中芦苇生长的影响[J]. 环境科学, 2015, 36(4)1481-1488.)
135. Guo Shaoxia, Liu Runjin. 2010. Effects of Arbuscular Mycorrhizal Fungi Glomus mosseae on Salt Tolerance of Paeonia suffruticosa Andr. *Plant Physiology Communications*,46 (10): 1007-1012.(With English abstract.郭绍霞, 刘润进. 丛枝菌根真菌Glomus mosseae对盐胁迫下牡丹渗透调节的影响[J]. 植物生理学报, 2010, 46(10): 1007-1012.)
136. Guo Yanni. 2015.The physiological response of different salt-tolerance Medicago Sativa L. colinized by arbuscular mycorrhiza fungi to salt stress. *Shanxi agricultural university*,master degree disseitation. (With English abstract.郭艳妮. 不同耐盐性苜蓿接种丛枝菌根真菌对盐胁迫的生理响应[D]. 山西农业大学, 2015.)
137. Han Bing, Guo Shirong, He Chaoxing, et al. 2012. Effects of arbuscular mycorrhiza fungi ( AMF) on the plant growth，fruit yield，and fruit quality of cucumber under salt stress. *Chinese Journal of Applied Ecology*, 23(1)154-158.(With English abstract. 韩冰, 郭世荣, 贺超兴,等. 丛枝菌根真菌对盐胁迫下黄瓜植株生长、果实产量和品质的影响[J]. 应用生态学报, 2012, 23(1)154-158.)
138. Han Bing, He Chaoxing, Guo Shirong, et al. 2011. Effects of arbuscular mycorrhizal fungi on osmoregulation substance contents and antioxidant enzyme activities of Cucumber seedlings under salt stress. *Acta Bot Borea*, 31(12):2492-2497. (With English abstract. 韩冰, 贺超兴, 郭世荣,等. 丛枝菌根真菌对盐胁迫下黄瓜幼苗渗透调节物质含量和抗氧化酶活性的影响[J]. 西北植物学报, 2011, 31(12):2492-2497.)
139. Han Tingting.2011. Mechanisms of Salt Tolerance Improved by Arbuscular Mycorrhizal Fungi in Paeonia suffruticosa. *Qingdao agricultural university*,Doctoral dissertation.(With English abstract. 韩婷婷. (2011). 丛枝菌根真菌提高牡丹耐盐性的机制.青岛农业大学,博士论文).
140. Ge Xueli, Zhao Lili, Li Yingpeng. 2005. Effects of AM fungi on the growth and protective enzymes of cotton under NaCI stress. *Acta ecologica sinica*, 25(1):188-193.(With English abstract.贺学礼, 赵丽莉, 李英鹏. NaCl胁迫下AM真菌对棉花生长和叶片保护酶系统的影响[J]. 生态学报, 2005, 25(1):188-193.)
141. He Zhongqun, He Chaoxing, Yan Yan, et al. 2011.Regulative Effect of Arbuscular Mycorrhizal Fungi on Water Absorption and Expression of Aquaporin Genes in Tomato Under Salt Stress*.Acta Horticulturae Sinica,*38(2):273-280.(With English abstract.贺忠群, 贺超兴, 闫妍,等. 盐胁迫下丛枝菌根真菌对番茄吸水及水孔蛋白基因表达的调控[J]. 园艺学报, 2011, 38(2):273-280.)
142. He Zhongqun, He Chaoxing.(2013).Effect of Arbuscular mycorrhizal Fungi on Nutrition Absorbing and Ion Damage in Tomato under Salt Stress. *Avta agriculturae boreali-sinica*, 28(1): 181-186. (With English abstract. 贺忠群, 贺超兴. 盐渍条件下丛枝菌根真菌对番茄营养吸收及离子毒害的影响[J]. 华北农学报, 2013, 28(1)181-186.)
143. He Zhongqun, Li Huanxiu, Tang Haoru,et al.2010.Effect of arbuscular mycorrhizal fungi on tomato endogenous under NaCl stress. *Journal of Nuclear Agricultural Sciences*, 24(5): 1099-1104. (With English abstract. 贺忠群, 李焕秀, 汤浩茹,等. 丛枝菌根真菌对NaCl胁迫下番茄内源激素的影响[J]. 核农学报, 2010, 24(5): 1099-1104.)
144. He Zhongqun, Zou Zhirong, He Chaoxing, et al. Effect of AMF on GSH-Px activity and cell membrane osmasis of tomato. *Journal of northwest sci-tech university of agriculture and forest*, 34(12): 53-57.(With English abstract. 贺忠群, 邹志荣, 贺超兴,等. 盐胁迫下丛枝菌根真菌对番茄细胞膜透性及谷光甘肽过氧化物酶活性的影响[J]. 西北农林科技大学学报自然科学版, 2006, 34(12):53-57.)
145. Hu Zhihong, Huang Jingxin,Du Shujia, et al.2010.Effects of arbuscularmycorrhizal fungi on plant seedling growth and nutrients uptaking under saline cond itions. *Journal of Shanghai Normal University (Natural Sciences)*,39(3):309-314.(With English abstract.胡志宏, 黄晶心, 杜书佳,等. 盐胁迫下丛枝菌根对植物幼苗生长和营养元素吸收的影响[J]. 上海师范大学学报(自然科学版), 2010, 39(3): 309-314.)
146. Huang Zhi, Xu Weiping, Yu Fangbin, et al. 2018. Photosynthesis responses of Cucumis melo seedlings to Glomus under low light and salt stress. *Acta Bot. Boreal-Occident. Sin*,3:0307-0315. (With English abstract. 黄志, 许炜萍, 郁昉斌,等. 接种AMF对弱光环境及盐胁迫下甜瓜光合特性的影响[J]. 西北植物学报, 2018(3):0307-0315).
147. Jia Tingting, Chang Wei, Fan Xiaoxu, et al. 2018. Effects of Arbuscular mycorrhizal fungi on photosynthetic and chlorophymm fluorescence characteristics in Elaeagnus angustifolia seedlings under salt stress. *Acta ecology sinica*, 38(4).(With English abstract. 贾婷婷, 常伟, 范晓旭,等. 盐胁迫下AM真菌对沙枣苗木光合与叶绿素荧光特性的影响[J]. 生态学报, 2018, 38(4).)
148. Li Lili. 2016. Research of the Effect of Dominant AM Fungi from Inula japonica Rhizosphere on Salt-alkali Tolerance of Trifolium repence.*Northeast Forestry University*, Dissertation for the Degree of Master.(With English abstract. 李丽丽. 旋覆花根围优势AM真菌对白花三叶草耐盐碱特性影响研究[D]. 东北林业大学, 2016.)
149. Li Tao, Yang Di,Yang Qing,et al.2008. Effects of Inoculation of Arbuscular Mycorrhizal Fungi Glomus mosseae on SOD and CAT Activities in Suaeda salsa Seedlings under Salt Stress.*Journal of Anhui Agri.Sci*, 36(35):15324-15325. (With English abstract. 李涛, 段迪, 杨青,等. 接种摩西球囊霉对盐胁迫条件下盐地碱蓬叶片SOD和CAT活性的影响[J]. 安徽农业科学, 2008, 36(35):15324-15325.)
150. Liu Hongguang.2016. Mechanisms of arbuscular mycorrhizal fungi (AMF) enhancing salt tolerance of Lycium Barbarum L.*Northwest A&F University*, Dissertation for Doctor Degree. (With English abstract. 刘洪光. AM真菌提高枸杞耐盐性的机制研究[D]. 西北农林科技大学, 2016.)
151. Liu Runjin, Wei Runjin, Kang Junshui. 1997.Effects of arbuscular mycorrhizal fungi on growth of lawn grasses grown in saline soil. *Journal of Laiyang Agricultural College*, 14(2):134-137. (With English abstract. 刘润进, 魏红, 康俊水. AM菌对盐渍化土壤中坪草生长的影响[J]. 莱阳农学报(自然科学版), 14(2):134-137.)
152. LiuXiaojie.2006.Study on Effects of Arbuscular Mycorrhizal Fungi on the Salt and Drought Resistance of Blackberry Cuttings.*Southwest University,* Dissertation for Master Degree.(With English abstract. 刘晓捷. 接种丛枝菌根真菌对黑莓扦插苗耐盐性以及抗旱性的影响[D]. 西南大学, 2006.)
153. Liu Jie, Xiao Bin, Wang Lixia, et al. 2013. Influence of AMF on salt tolerance of tea. *Journal of Northwest A&F university(Nat.Sci.Ed.)*, 42(3)220-226. (With English abstract. 柳洁, 肖斌, 王丽霞,等. 丛枝菌根真菌对茶树耐盐性的影响[J]. 西北农林科技大学学报自然科学版, 2014, 42(3)220-226.)
154. Liu Jie, Xiao Bin, Wang Lixia, et al. 2013. Influence of AM on the Growth of Tea Plant and Tea Quality under Salt Stress. *Journal of Tea Science*, (2):140-146. (With English abstract. 柳洁, 肖斌, 王丽霞,等. 盐胁迫下丛枝菌根(AM)对茶树生长及茶叶品质的影响[J]. 茶叶科学, 2013, (2): 140-146.)
155. Lu Shuang, Guo Huan, Wang Shaoming, et al.2011. Effects of AM Fungi on Growth and Physiological Characters of Medicago sativa L. under NaCl Stress. *Journal of Soil and Water Conservation*, 25(2):227-231.(With English abstract. 陆爽, 郭欢, 王绍明,等. 盐胁迫下AM真菌对紫花苜蓿生长及生理特征的影响[J]. 水土保持学报, 2011, 25(2):227-231.)
156. Ma Pengkun. 2016.Effects of arbuscular mycorrhizal fungi on the growth of Suaeda glauca in the soil from saline-alkaline wetlands.*Inner Mongolia University,* Dissertation for Master Degree.(With English abstract. 马朋坤. 丛枝菌根真菌对盐碱化湿地土壤上碱蓬生长的影响研究[D]. 内蒙古大学, 2016.)
157. Ma Yabin, Li Wei, Xu Meng, et al.2014. Effects of arbuscular mycorrhiza fungi on growth and photosynthesis of Lily under salt stress.*Journal of Qingdao agrocultural university (Natural Science)*, (3):157-161. (With English abstract. 马亚斌, 李伟, 徐萌,等. AM 真菌对盐胁迫下百合生长和光合作用的影响[J]. 青岛农业大学学报(自然科学版), 2014, (3):157-161.)
158. Nan Xueqin. 2016. Effects of different arbuscular mycorrhiza fungus on the growth and salt tolerance of tea plant. *Northwest A&F University*,Professional Master's Degree. (With English abstract. 南雪芹. 不同丛枝菌根真菌(AMF)对茶树生长及耐盐性的影响研究[D]. 西北农林科技大学, 2016.)
159. Shen Lianying, Mao Yongmin, Lu Jinying, et al. 2004.Effects of arbuscular mycorrhizea on salt tolerance of wild Jujube (Zizyphus spinosus hu) seedling. *Acta oedologica sinica*, 41(3):426-433. (With English abstract. 申连英, 毛永民, 鹿金颖,等. 丛枝菌根对酸枣实生苗耐盐性的影响[J]. 土壤学报, 2004, 41(3):426-433.)
160. Sheng Min, Tang Min, Zhang Fengfeng, et al.2011. Effect of AM Fungi on Salt Resistance of Maize. *Acta Bot. Boreal. - Occident. Sin*. 31(2): 332-337. (With English abstract. 盛敏, 唐明, 张峰峰,等. 盐胁迫下接种AM真菌对玉米耐盐性的影响[J]. 西北植物学报, 2011, 31(2): 332-337.）
161. Sun Yufang, Song Fuqiang, Chang Wei, et al. 2016.Effect of Arbuscular Mycorrhizal Fungi on Growth and Physiology of Elaeagnus angustifolia Seedlings Subjected to Salinity Stress. *Scientia silvae sinicae*, 52(6):18-28. (With English abstract. 孙玉芳, 宋福强, 常伟,等. 盐碱胁迫下AM真菌对沙枣苗木生长和生理的影响[J]. 林业科学, 2016, 52(6):18-28.)
162. Sun Yuechun, Chen Xin, Yin Kuide.2011. Effects of Arbuscular Mycorrhizal Fungi on Arctium lappa L. Seedling Growth under Saline Stress. *Heilongjiang Agricultural Sciences*, (6): 93-96. (With English abstract. 孙跃春, 陈鑫, 殷奎德. 盐胁迫下接种丛枝菌根真菌对牛蒡幼苗生长的影响[J]. 黑龙江农业科学, 2011 (6): 93-96.)
163. Tang Jian. 2015. Effeets of AMF on Salt Tolerance of ,Sedum aizoon L Under NaCI Stress. *Sichuan Agricultural University*, professional Master's Degree.(With English abstract. 唐剑. NaCl胁迫下丛枝菌根真菌(AMF)对养心菜耐盐性的影响[D]. 四川农业大学, 2015.)
164. Wang Bin, Yao Zhaoqun, Zhao Sifeng, et al.2013.Effect of AM Fungi on processing Tomato seedlings physiological characteristics and AVPI gene expression under salt stress. *Acta Bot. Boreal. - Occident. Sin.,* 33(10): 2016-2023. (With English abstract. 王斌, 姚兆群, 赵思峰,等. AM真菌对盐胁迫下番茄幼苗生理特征及AVP1表达的影响[J]. 西北植物学报, 2013, 33(10): 2016-2023.)
165. Wang Na, Chen Fei, Yue Yingnan and Yang Chunxue. 2017. Effects of two dominant arbuscular mycorrhizal fungi on salt tolerance of alfalfa in Songnen saline-alkaline grassland. *Jiangsu agriculture science*, 45(24), 146-148.(With Chinese abstract. 王娜, 陈飞, 岳英男, & 杨春雪. (2017). 松嫩盐碱草地2种优势丛枝菌根真菌对紫花苜蓿耐盐性的影响. 江苏农业科学, 45(24), 146-148.)
166. Wang Yiming, Tang Jian, Long Shengjv,et al. 2017.Effect of AMF on growth and chlorophyII fluorescence parameters of Sedum aizoon L. seedlings under NaCl stress. *Agricultural Research in the Arid Areas*, 35(6): 134-139. (With English abstract. 王一鸣, 唐剑, 龙胜举,等. NaCl胁迫对接种AMF费菜生长和叶绿素含量及荧光参数的影响[J]. 干旱地区农业研究, 2017, 35(6): 134-139.)
167. Wu Qiangsheng, Liu Wei, Zhai Huafen, et al.2010. Influences of AM Fungi on Growth and Root Antioxidative Enzymes of Trifoliate Orange Seedlings under Salt Stress. *Acta Agriculturae Universitatis Jiangxiensis*, 32(4): 759-763. (With English abstract. 吴强盛, 柳威, 翟华芬,等. 盐胁迫下AM真菌对枳实生苗生长和根系抗氧化酶的影响[J]. 江西农业大学学报, 2010, 32(4):759-763.)
168. Xie Xiaohong. 2016. Effects of Arbuscular Mycorrhizal Fungi on Growth and Photosynthesis in Melon Seedlings under Weak Light with Salt Stress.*Sichuan Agricultural University, professional Master's Degree.* (With English abstract. 谢晓红. 丛枝菌根真菌对弱光及盐胁迫下甜瓜生长和光合作用的影响[D]. 四川农业大学, 2016.)
169. Xu Yao, Fan Yan, Yu Yunhe, et al. 2014.Effects of arbuscular mycorrhizal fungus on the growth and physiological salt tolerance parameters of Carthamus tinctorius seedlings under salt stress. *Chinese Journal of Ecology*, 33(12): 3395-3402. (With English abstract. 徐瑶, 樊艳, 俞云鹤,等. 丛枝菌根真菌对盐胁迫下红花幼苗生长及耐盐生理指标的影响[J]. 生态学杂志, 2014, 33(12): 3395-3402.)
170. Yan Zhichen, Li Yingde, Cheng Weijia, et al.2018. Effects of AM fungi and grass endophyte on the growthofsyegrass under different salt concentrations.*Grassland and Turf*, 38(1), 63-70. (With English abstract. 闫智臣, 李应德, 程维佳,等. 不同盐浓度下AM真菌和禾草内生真菌对多年生黑麦草生长的影响[J]. 草原与草坪, 2018,38(1),68-70.)
171. Yang Haixia, Li Shixia, Guo Shaoxia. 2014.Effects of Arbuscular Mycorrhizal Fungi on Salinity Tolerance of Lagerstroemia indica. *Plant Physiology Journal*, 50 (9): 1379~1386. (With English abstract. 杨海霞, 李士美, 郭绍霞. 丛枝菌根真菌对紫薇耐盐性的影响[J]. 植物生理学报, 2014,50(9):1379-1387.)
172. Yang Haixia, Li Shixia, Li Min, et al.2014. Effects of arbuscular mycorrhizal fungi on salinity tolerance of Trifolium repens L.*Journal of Qingdao agricultural university (Natural science)*, 31(2): 85-90. (With English abstract. 杨海霞, 李士美, 李敏,等. 丛枝菌根真菌对白三叶耐盐性的影响[J]. 青岛农业大学学报(自然科学版), 2014, 31(2): 85-90.)
173. Yang Haixia, Liu Runjin, Guo Shaoxia. 2014. Effcts of arbuscular mycorrhizal fungus Glomus mosseaeon the growth characteristics of Festuca arundinacea under salt stresss conditions.*Acta prataculturae sinica*,23(4):195-203.(With English abstract. 杨海霞, 刘润进, 郭绍霞. AM真菌摩西球囊霉对盐胁迫条件下高羊茅生长特性的影响[J]. 草业学报, 2014, 23(4):195-203.)
174. Yang Haixia, Xu Meng, Liu Ning, et al. 2014. Effects of arbuscular mycorrhizal fungi on salinity tolerance of two turfgrss.*Pratacultural science*, 31(7): 1261-1268.(With English abstract. 杨海霞, 徐萌, 刘宁,等. 丛枝菌根真菌对两种草坪草耐盐性的影响[J]. 草业科学, 2014, 31(7): 1261-1268.)
175. Yang Ruihong, Liu Runjin, Liu Liancheng, et al.2009. Effects of Arbuscular Mycorrhizal Fungi and Salicylic Acid on Salt Tolerance of Strawberry (Fragaria×ananassa Duch) Plants. *Scientia Agricultura Sinica*,42(5): 1590-1594. (With English abstract. 杨瑞红, 刘润进, 刘成连,等. AM真菌和水杨酸对草莓耐盐性的影响[J]. 中国农业科学, 2009, 42(5): 1590-1594.)
176. Yue Yingnan. 2015.Effects of arbuscular mycorrhizal funi on the salt tolerance of plants in saline-alkaline grassland of Songnen plain. *Northeast forestry university*, Doctoral dissertation. (With English abstract. 岳英男. (2015). 松嫩盐碱草地主要丛枝菌根真菌对植物耐盐性影响的研究. 东北林业大学,博士学位论文)
177. Zai Xue Ming, Hao Shanshan, Shao Zhiguang, et al.2013. Effects of Glomus sinensis on Chlorophyll Content and Fluorescence Parameters of Bamboo Shoots under NaCl Stress. *Jiangsu agriculture science*, (41(8):175-178.(With Chinese abstract. 宰学明, 郝姗姗, 邵志广,等. 摩西球囊霉对NaCl胁迫下竹柳苗叶绿素含量和荧光参数的影响[J]. 江苏农业科学, 2013, 41(8):175-178.)
178. Zai Xueming, Hao Zhenping, Zhang Huanshi, et al.2013.Effects of AM Fungi on Ascorbate-Glutathione Cycle Metabolism in Leaves of Prunus maritima Marshall under NaCl Stress. *Plant Physiology Journal*, 4(9(1):41-46. (With English abstract. 宰学明, 郝振萍, 张焕仕,等. NaCl胁迫下AM真菌对滨梅叶片中抗坏血酸-谷胱甘肽循环的影响[J]. 植物生理学报, 2013, 49(1):41-46.)
179. Zhang Hua, Bao Yuying, Tebuqin. Effect of Arbuscular Mycorrhizal Fungi on Growth and the Total Flavonoids of Caragana microphylla Lam. under Salt Stress. *Advance in Microbiology*, 02(3):71-77. (With English abstract. 张华, 包玉英, 特布沁. 盐胁迫下接种丛枝菌根真菌(AMF)对小叶锦鸡儿的生长及总黄酮含量的影响[J]. Advances in Microbiology, 2013, 02(3):71-77.)
180. Zhang Y F，Wang P，Bi Q，Zhang Z H，Yang Y F.(2017). The effect of the arbuscular mycorrhizal fungi on the growth of Leymus chinensis under saline stress of different intensities. *Acta Ecologica Sinica*, 36(17) : 5467-5476. (With English abstract. 张义飞, 王平, 毕琪,等. 不同强度盐胁迫下AM真菌对羊草生长的影响[J]. 生态学报, 2016, 36(17):5467-5476.)
181. Zhao Qi, Bao Yuying. 2015. Effect of Arbuscular mycorrhizal fungi on growth and two phenolic acids of Medicago sativa under various mixed salt-alkaline stresses. *Acta Bot. Boreal.-Occident. Sin.*, 35(9):1829-1836. (With English abstract. 赵琦, 包玉英. 混合盐碱胁迫下丛枝菌根真菌对紫花苜蓿生长及2种酚酸含量的影响[J]. 西北植物学报, 2015, 35(9):1829-1836.)
182. Zhao Xia, Ye Lin, Na Xuewei, et al. 2017. Influence of arbuscular mycorrhizal fungus on the osmotic adjustment substance and antioxidant system of Medicago sativa under salt-alkaline stress. *Jiangsu J. of Agr. Sci.*,33(4):782-787. (With English abstract. 赵霞, 叶林, 纳学伟,等. 盐碱胁迫下丛枝菌根真菌对紫花苜蓿渗透调节物质及抗氧化能力的影响[J]. 江苏农业学报, 2017, 33(4):782-787.)

**Table S1 Detailed information of plant salt tolerant classification and plant species**

| **Plant categories** | **Families** | **Plant species** | **Percent** |
| --- | --- | --- | --- |
| Glycophytes(813) | Fabaceae (209) | *Acacia auriculaeformis* (9), *Acacia gerrardii Benth* (2), *Acacia nilotica* (3), *Arachis hypogaea L*.(4), *Astragalus sinicus L*.(12), *Cajanus cajan L.*(19), *Vigna unguiculata* (4), *Glycine max (L.) Merr.*(6), *Phaseolus vulgaris L*.(13), *Phaseolus L*.(12), *Trifolium repens L*.(21), *Trigonella foenum-graecum L*.(9), *Medicago sativa L*.(70), *Pisum sativum L*.(3), *Sesbania aegyptiaca* (1), *Sesbania grandiflora* (1), *Robinia pseudoacacia L*.(4), *Lupinus termis Forsik* (1), *Cicer arietinum* (15) | 88.76% |
|  | Poaceae (185) | *Bromus inermis Leyss*.(6), *Brachiaria humidicola* (4), *Festuca arundinacea* (13), *Hordeum vulgare L*.(6), *Oryza glaberrima* (3), *Triticum aestivum L.*(50), *Sorghum bicolor L*.(6), *Zea mays L.*(79), *Pennisetum glaucum* (6), *Poa paratensis* (9), *Lolium perenne L*.(3) |  |
|  | Liliaceae (12) | *Allium cepa L.* (6), *Lilium brownii* (6) |  |
|  | Asparagaceae (2) | *Asparagus officinalis L*. (2) |  |
|  | Asteraceae (67) | *Arctium lappa L*. (18), *Chrysanthemum morifolium (Ramat.) Tzvel*. (6), *Lactuca sativa L*. (34),  *Carthamus tiuctorius L*. (9) |  |
|  | Scrophulariaceae (8) | *Bacopa monnieri L.* (8) |  |
|  | Brassicaceae (6) | *Brassica juncea L*. (3), *Brassica napus L*. (3) |  |
|  | Theaceae (13) | *Camellia sinensis L.* (13) |  |
|  | Solanaceae (62) | *Capsicum annuum L*. (16), *Lycopersicon esculentum L*. (46) |  |
|  | Cucurbitaceae (7) | *Cucumis melo L*. (4), *Cucumis sativus L*. (3) |  |
|  | Rosaceae (67) | *Fragaria×ananassa Duch., Rubus L*. (34), *Malus pumila Mill* (9), *Prunus maritima* (3),  *Pynus betulifolia Bge* (12), *Rubus L.* (9) |  |
|  | Lamiaceae (30) | *Mentha arvensis L*. (5), *Ocimum basilicum L*. (25) |  |
|  | Musaceae (12) | *Musa nana Lour*. (12) |  |
|  | Salicaceae (3) | *Salix babylonica* (1), *Populus cathayana Rehd* (2). |  |
|  | Paeoniaceae (15) | *Paeonia suffruticosa* (15) |  |
|  | Lythraceae (3) | *Lagerstroemia indica* (3) |  |
|  | Anacardiaceae (20) | *Sclerocarya birrea* (2), *Pistacia vera L.* (18). |  |
|  | Crassulaceae (3) | *Kalanchoe blossfeldiana Poelin* (3) |  |
|  | Vitaceae (2) | *Vitis vinifera L.* (2) |  |
|  | Malvaceae (34) | *Gossypium arboreum L*. (34) |  |
|  | Rutaceae (27) | *Carrizo citrange* (1), *Citrus L.* (4)., *Citrus macrophylla Wester*. (1)*，Citrus sinensis×Poncirus trifoliata.*(3), *Cleopatra mandarin* (1), Poncirus *trofoliata L.* (15), *Pineapple sweet orange* (1), *Sour orange* (1) |  |
|  | Ephedraceae (2) | *Ephedra aphylla Forssk* (2) |  |
|  | Rhamnaceae (12) | *Ziziphus spina-christi* (6), *Ziziphus jujuba Mill*. (6) |  |
|  | Euphorbiaceae (12) | *Jatropha carcas L.* (12) |  |
| 1. Halophytes (103) | Asteraceae (8) | *Asteriscus maritimus* (8) | 1. 11.24% |
|  | Chenopodiaceae (20) | *Atriplex nummularia* (4), *Suaeda physophora* (8), *Suaeda salsa* (8) |  |
|  | Fabaceae (4) | *Caragana microphylla Lam.* (4) |  |
|  | Poaceae (35) | *Danthonia spicata* (4), *Phragmites australis* (10), *Leymus chinensis* (14), *Panicum turgidum Forssk* (1), *Puccinellia tenuiflora* (6) |  |
|  | Crassulaceae (16) | *Sedum aizoon L.* (16) |  |
|  | Elaeagnaceae (9) | *Elaeagnus angustifolia Linn.* (9) |  |
|  | Malvaceae (9) | *Kostelelzkya virginica* (9) |  |
|  | Solanaceae (2) | *Lycium barbarum L*. (2) |  |

**Table S2** **Rank correlation tests for publication bias and fail-safe numbers**

| Categories | Sample size | Effect size (95% CI) | Spearman’s rank order correlation | | Fail-safe numbers |
| --- | --- | --- | --- | --- | --- |
|  |  |  | R | P |  |
| K^+^/Na^+^ ratio | 272 | 0.33 (0.23 to 0.41) | 0.088 | 0.146 | 833 865 |
| Ca^2+^/Na^+^ ratio | 145 | 0.21 (0.07 to 0.32) | 0.034 | 0.687 | 94 406 |
| Total chlorophyll | 282 | 0.34 (0.3 to 0.38) | 0.017 | 0.777 | 9 791 692 |
| Root total length | 33 | 0.36 (0.26 to 0.47) | -0.068 | 0.707 | 12 597 |
| Root tips number | 25 | 0.54 (0.31 to 0.89) | 0.267 | 0.196 | 383 |
| Root volume | 34 | 0.49 (0.38 to 0.6) | 0.050 | 0.781 | 24 361 |
| Root surface area | 36 | 0.47 (0.39 to 0.56) | 0.060 | 0.729 | 25 246 |





**Figure S1 Relationships between effect size of SOD activities (a), CAT activities (b) and effect size of MDA content in plants after AMF inoculation under salt stress conditions.** Blue dots indicate the values in mycorrhizal halophytes, and pink dots indicate the values in mycorrhizal glycophytes.





**Figure S2 Relationships between effect sizes of N uptake (a), P uptake (b), K^+^ uptake (c), MDA content (d) and effect sizes of chlorophyll content in plants after AMF inoculation under salt stress conditions.**
